# Continuous high-frequency deep brain stimulation of the anterior insula modulates autism-like behavior in a valproic acid-induced rat model

**DOI:** 10.1186/s12967-022-03787-9

**Published:** 2022-12-06

**Authors:** Lifei Xiao, Shucai Jiang, Yangyang Wang, Caibin Gao, Cuicui Liu, Xianhao Huo, Wenchao Li, Baorui Guo, Chaofan Wang, Yu Sun, Anni Wang, Yan Feng, Feng Wang, Tao Sun

**Affiliations:** 1 Ningxia Key Laboratory of Cerebrocranial Disease, Incubation Base of National Key Laboratory, Ningxia Medical University, Yinchuan, 750000 China; 2 Department of Neurosurgery, Weifang People’s Hospital, Weifang, 261000 China; 3 Department of Neurosurgery, General Hospital of Ningxia Medical University, Yinchuan, 750000 China; 4 Department of Otolaryngology and Head Surgery, The First People’s Hospital of Yinchuan, Yinchuan, 750000 China; 5 Department of Neurosurgery, Shaanxi Provincial People’s Hospital, Xi’an, 710000 China; 6 Department of Neurosurgery, The First Affiliated Hospital, Zhejiang University School of Medicine, Hangzhou, 310000 China

**Keywords:** Autism spectrum disorder, Deep brain stimulation, Insula cortex, Valproic acid, Proteomics

## Abstract

**Background:**

Until now, the treatment of patients with autism spectrum disorder (ASD) remain a difficult problem. The insula is involved in empathy and sensorimotor integration, which are often impaired in individuals with ASD. Deep brain stimulation, modulating neuronal activity in specific brain circuits, has recently been considered as a promising intervention for neuropsychiatric disorders. Valproic acid (VPA) is a potential teratogenic agent, and prenatal exposure can cause autism-like symptoms including repetitive behaviors and defective sociability. Herein, we investigated the effects of continuous high-frequency deep brain stimulation in the anterior insula of rats exposed to VPA and explored cognitive functions, behavior, and molecular proteins connected to autism spectrum disorder.

**Methods:**

VPA-exposed offspring were bilaterally implanted with electrodes in the anterior insula (Day 0) with a recovery period of 1 week. (Day 0–7). High-frequency deep brain stimulation was applied from days 11 to 29. Three behavioral tests, including three-chamber social interaction test, were performed on days 7, 13, 18, 25 and 36, and several rats were used for analysis of immediate early genes and proteomic after deep brain stimulation intervention. Meanwhile, animals were subjected to a 20 day spatial learning and cognitive rigidity test using IntelliCage on day 11.

**Results:**

Deep brain stimulation improved the sociability and social novelty preference at day 18 prior to those at day 13, and the improvement has reached the upper limit compared to day 25. As for repetitive/stereotypic-like behavior, self- grooming time were reduced at day 18 and reached the upper limit, and the numbers of burried marbles were reduced at day 13 prior to those at day 18 and day 25. The improvements of sociability and social novelty preference were persistent after the stimulation had ceased. Spatial learning ability and cognitive rigidity were unaffected. We identified 35 proteins in the anterior insula, some of which were intimately linked to autism, and their expression levels were reversed upon administration of deep brain stimulation.

**Conclusions:**

Autism-like behavior was ameliorated and autism-related proteins were reversed in the insula by deep brain stimulation intervention, these findings reveal that the insula may be a potential target for DBS in the treatment of autism, which provide a theoretical basis for its clinical application., although future studies are still warranted.

**Supplementary Information:**

The online version contains supplementary material available at 10.1186/s12967-022-03787-9.

## Introduction

Autism spectrum disorder (ASD) is a neurodevelopmental disease that is both prevalent and heterogeneous and is hallmarked by impairments in social interaction and communication, behaviors that are very repetitive and restricted interest, and/or sensory-motor sensitivity and behaviors, which results in enormous cognitive, economic, and medical challenges [1–3]. The causative factors of ASD mainly include environmental and genetic factors. Prenatal and perinatal factors such as maternal diet and lifestyle have been widely reported and described, as well as advanced parental age, maternal obesity, gestational diabetes mellitus, and prenatal drug exposure (such as valproate use during pregnancy) [1, 4]. Although early psychological/behavioral interventions, complementary therapy, or drug treatments can address some autism symptoms in children, such as reducing anxiety, aggression, repetitive stereotyped behavior, and the combined symptoms of inattention and hyperactivity, there is still no definitive treatment for the disorder [5]. Therefore, additional approaches are needed to improve individuals’ quality of life and enhance the efficacy of current strategies.

In recent decades, neuromodulation technology has attracted more attention, especially deep brain stimulation (DBS), which is a neurosurgical treatment that is adjustable, reversible, and minimally invasive. Although the mechanisms of DBS are currently not unified and clear, it can be determined that DBS has complex electrical effects on individual neurons and neuronal networks, altering neurotransmitter concentrations and dynamics, and influencing the microenvironment including astrocytes, microglia, and endothelial cells [6]. DBS also affects neuroplasticity and may induce neuroprotection and neurogenesis [7]. At present, a popular treatment option for a wide range of refractory neurological and psychiatric conditions, including obsessive-compulsive disorder, depression, chronic pain, tremors, dystonia, and Parkinson’s disease, is DBS [8–11]. Some preclinical studies have shown that DBS can address certain autism-like behaviors, such as increasing social interaction and suppressing repetitive behaviors [12–14]. However, the optimal target of stimulation remains to be further explored.

The insula has long been an underestimated area of the brain. Recently, mounting research evidence has shown that the insula is a hub that has a substantial connection to a vast network of cortical and subcortical brain areas, which assume an instrumental function in social and non-social functions such as emotion, motivation, perception, empathy, risk assessment, decision-making, and sensorimotor integration [15, 16]. At the neural network level, previous studies have shown that the insula is a key interface of the “salience network” in human and animal brains, which has been linked to the recognition and integration of both sensory and emotional stimuli, as well as being responsible for operating the switch that toggles between the inwardly oriented cognition of the “default mode network” and the outwardly oriented cognition of the “central executive network” [17, 18]. An increasing body of neuroimaging studies has shown that young individuals diagnosed with high-functioning autism present reduced volume and thickness in the posterior and anterior regions of the insula [19]. Moreover, hypo-connectivity between the insula, cuneus, and superior marginal gyrus in autistic children has been identified, and there is hyperconnectivity between the insula and superior temporal gyrus [20]. The insula has been demonstrated in several recent behavioral investigations of pharmacological and chemical treatments to play a role in parts of the brain’s processing of socioemotional information as well as pain [21–24]. For example, oxytocin receptors in the insular cortex exert a fundamental function in both asocial and prosocial responses to stressed conspecifics, and intra-insular oxytocin administration may regulate social-emotional behaviors [22].

The antiepileptic drug valproic acid (VPA) is a potential teratogen that has been linked to higher rates of autism and birth defects following prenatal exposure [25]. In rodents, prenatal VPA exposure gives rise to autism-like symptoms including repetitive behaviors and defective sociability, which has been extensively demonstrated in studies of autism [23, 26]. Considering the complexity of insular functions and encouraging the results of preclinical and clinical studies of DBS interventions in psychiatric disorders, This research examined the influence of continuous DBS in the anterior insula (AI) on autistic-like behavior in a rat model that had been treated with valproic acid. In addition, we further applied a label-free proteomic analysis to detect the AI proteins that were modulated by the DBS treatment. This study may supplement the relevant basic database of ASD animal models and provide a basis and direction for the effects and mechanisms of DBS in the treatment of ASD.

## Materials and methods

### Animals

The Animal Center of Ningxia Medical University provided the Sprague Dawley rats that ranged in weight from 230 to 250 g. The rats were kept in an environment with a temperature that was adjusted to between 22 ± 2 °C, with four to five rodents housed in each cage and a light/dark cycle of 12 h on, 12 h off, with free access to water and food at all times. All behavioral trials were conducted between the hours of 9:00 and 17:00. All animal experiments and care procedures were carried out in compliance with the guidelines of the National Institute of Health Guide for the Care and Use of Laboratory Animals and were approved by the Institutional Animal Care and Use Committee of Ningxia Medical University [IACUC Animal Use Certificate No.: SCXK (Ning) 2019–152]. Every experiment was designed to minimize the suffering of the animals and decrease the total number of animals involved.

## The rat model of autism induced by valproic acid

To avoid maternal death and miscarriages, the rat model has been approved in previous studies [23, 24]. A solution of 200 mg/ml with a pH of 7.3 was prepared by dissolving the sodium salt of valproic acid (NaVPA; P4543, Sigma-Aldrich) in saline at a concentration of 0.9%. On the 12.5th day of embryonic development, pregnant female SD rats were intraperitoneally injected with a single dose of 500 mg/kg valproic acid, whereas those in the control group were administered an identical dosage of 0.9% saline. On postnatal day 23, the rats were weaned, and after that, rats of both sexes were kept individually in cages that could hold four to five rats each. Follow-up experiments were performed on six-week-old VPA-exposed and male saline-exposed rats.

## Animal groups and experimental protocols

Experimental rats for measuring autism-like related behavior were assigned to six distinct categories (groups): (1) the saline group: the saline-exposed rats without the DBS system implantation (n = 13 for Experiment 1), (2) the VPA group: the VPA-exposed rats without the DBS system implantation (n = 12 for experiment 1), (3) the saline-sham group: the Saline-exposed rats were implanted with the DBS system but had no active stimulation (n = 13 for Experiment 1), (4) the VPA-sham group: the VPA-exposed rats were implanted with the DBS system but had no active stimulation (n = 12 for Experiment 1), (5) the saline-DBS group: the saline-exposed rats were implanted with the DBS system and had continuous electrical stimulation (n = 13 for Experiment 1), (6) the VPA-DBS group: the VPA-exposed rats were implanted with the DBS system and had continuous electrical stimulation (n = 14 for Experiment 1). To measure spatial learning and cognitive rigidity, experimental rats were classified into five distinct groups: (1) the saline group (n = 5 for Experiment 2), (2) the VPA group (n = 5 for Experiment 2), (3) the saline-sham group (n = 5 for Experiment 2), (4) the VPA-sham group (n = 5 for Experiment 2), (5) the VPA-DBS group (n = 5 for Experiment 2). The samples required for real-time quantitative polymerase chain reaction (RT-qPCR) analysis and proteomics analysis were collected from the same animals in the following groups: (1) the saline group (n = 3), (2) the VPA group (n = 3), (3) the saline-DBS group (n = 3), (4) and the VPA-DBS group (n = 3).

**Fig. 1:**
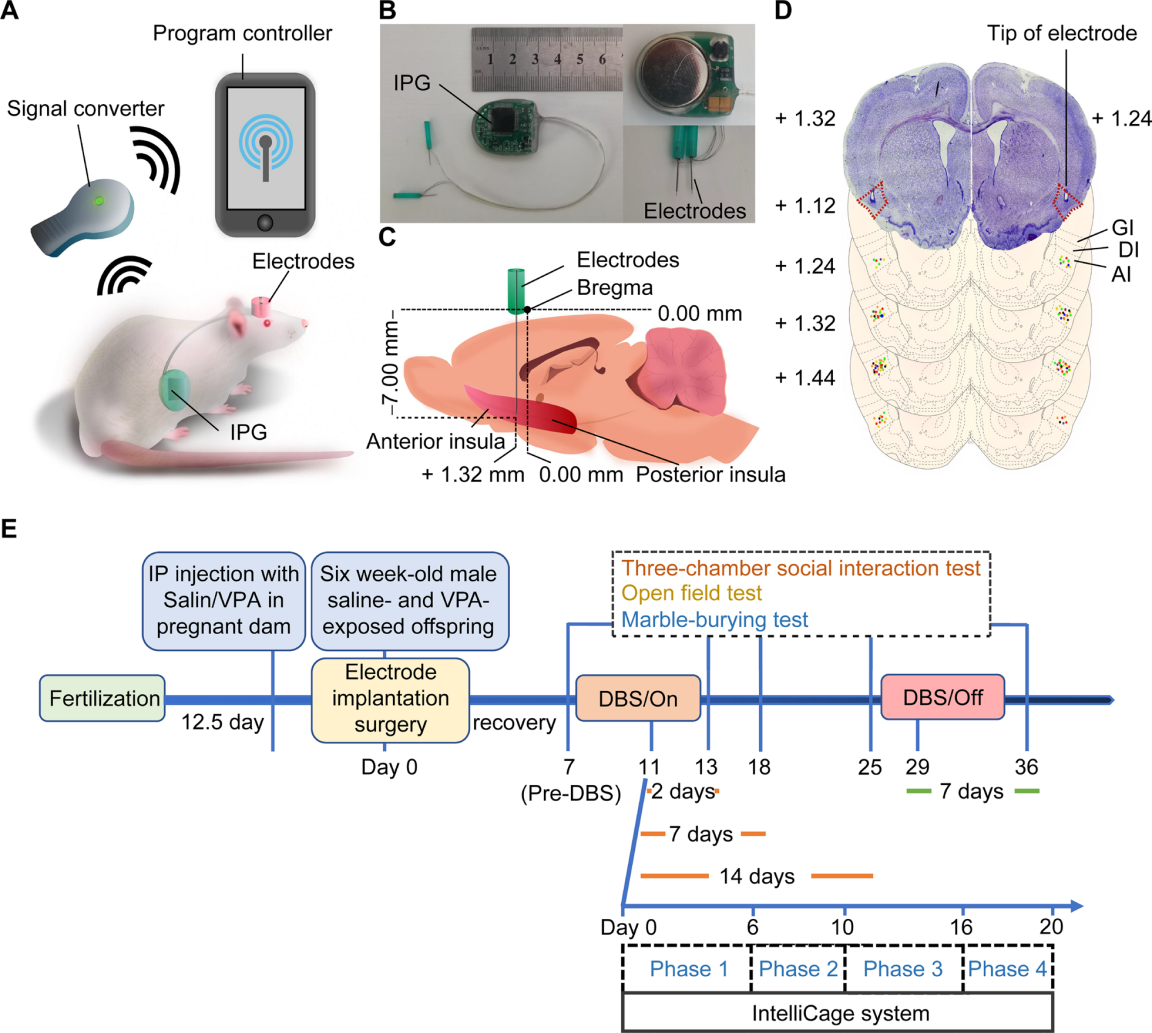
Deep brain stimulation system and electrodes implantation in rat anterior insula. **A** Electrical stimulation pattern of bilateral insular cortex in rats. Program controller was used to adjust parameters of IPG through the signal converter. **B** Photograph of the DBS apparatus consisting of an implantable pulse generator (IPG) and two electrodes. **C** A schematic view of the sagittal rat brain section showing the target of electrode implantation. **D** Targets of implanted electrodes (colored dots) in the AI of rats included for behavioural data analysis (n = 67). Experiment 1: saline-sham (green), n = 13; saline-DBS (yellow), n = 13; VPA-sham (orange), n = 12; VPA-DBS (red), n = 14. Experiment 2: saline-sham (blue), n = 5; saline-DBS (purple), n = 5; VPA-DBS (black), n = 5. **E** Experimental timeline. Six-week-old male saline- and VPA-exposed offspring were bilaterally implanted with DBS system (Day 0) followed by a 7 day recovery. HF-DBS (120 Hz, 150 µA, 90 µs) was continuously applied for 18 days (Day 11–29) and turned off on day 29. The autism-like related behavioural tests were performed on day 7, 13, 18, 25 and 36. The spatial learning and cognitive rigidity tests were performed on day 11 for 20 days. *IPG* implantable pulse generator, *GI* granular insular cortex, *DI* dysgranular insular cortex, *AI* agranular insular cortex. The schematic diagrams of rat brain coronal sections were adapted from the atlas of Paxinos and Watson [27]

***Experiment 1***: The experimental protocols used in this experiment aimed to examine the impact of DBS on autism-like behavior in the VPA-exposed rats (Fig. 1E). After bilaterally implanting DBS electrodes into the insula of six-week-old rats (Day 0), the animals were subjected to recovery from the procedure for seven days. The autism-like related behavior tests were conducted on days seven (pre-DBS), 13 (DBS for two days), 18 (DBS for seven days), 25 (DBS for 14 days), and 36 (DBS-off for seven days). In the saline-DBS and VPA-DBS groups, electrical stimulation was turned on on day 11 and turned off on day 29. The saline-sham and VPA-sham groups received surgery although there was no active stimulation at any point throughout the period. Rats in both the VPA and saline groups were not subjected to any surgical procedures or stimulation. Behavior tests included a three-chamber social interaction test, an open field test, a marble burying test.

***Experiment 2:*** The experimental protocols used in this experiment aimed to study the effects of DBS on spatial learning, exploratory power, and cognitive rigidity in VPA-exposed rats (Fig. 1E). After bilaterally implanting DBS electrodes into the insula of 6-week-old rats (on day 0), the animals were subjected to recovery from the procedure for seven days. Spatial learning and cognitive rigidity tests were performed on day 11 (stimulation-on). In the saline-DBS and VPA-DBS groups, electrical stimulation was turned on day 11 until the behavioral experiments were completed. Rats in the saline-sham group received surgery, but they were not given any active stimulation at any point throughout the period. Rats in groups that were given saline and VPA were not subjected to any surgical procedures or stimulation. The test design included four phases using the IntelliCage system (TSE Systems GmbH, Germany), as shown in “Behavioral tests”.

## Electrodes implantation and stimulation

The DBS apparatus (Beijing PINS Medical Co., Ltd., Beijing, China) used in our study was designed premised on a clinically applied DBS therapy system, which included an electrical stimulation device that could be implanted as well as a programmer that was fitted externally. The former contained implantable pulse generators (IPG), an extension lead, and coaxial electrodes, while the latter was comprised of a signal converter and program controller (Fig. 1A, B). The total length of the electrode was approximately 10 mm, which consisted of a stainless-steel outer tube (with a diameter of 0.3 mm) and its inner core (a diameter of 0.2 mm), and an insulating coating of perylene was applied to the contact area between them. The outer tube served as the reference pole, and the inner core's uncoated tip acted as the negative polarity's stimulating pole. The IPG was made up of an electrode connection, a button-type battery, and a microprocessor circuit board, which was connected to the extension lead and insulated by a layer of perylene. The lead extension constituted of a flexible platinum-iridium wire that had an insulating coating applied to it. This wire was connected to the electrode connection port of the pulse generator as well as the end of the electrode. The entire implantable DBS system weighed less than seven grams (5–7 g) (Fig. 1B).

The seal integrity and battery voltage of the electrodes were tested before surgery, and the qualified products were used for our experiments. Inhalation of isoflurane (4 percent induction, 2 percent maintenance) was used to put the rats under anesthesia before they were fixed on a stereotaxic device. A single dosage of penicillin (30,000 units, injected intramuscularly) was administered to the rats. Before any surgical operations were performed, the incision sites were injected with a 0.5% local anesthetic Novocain. A heating device was used to maintain the rats' body temperature at 37 °C. The IPG was surgically placed beneath the surface of the back skin, and the electrodes and extension lead pierced the skin of the neck through the subcutaneous tunnel and exposed to the head (Fig. 1A). Two holes, each measuring 2 mm in diameter and corresponding to the target region of the bilateral AI, were drilled into the skull of the rats till the dura mater was visible, and a total of four screws made of stainless steel were used to fasten the exposed skull. Subsequently, electrodes were surgically inserted bilaterally into the AI based on the coordinates (relative to bregma: anteroposterior  + 1.32 mm, lateral ± 5.00 mm, and dorsoventral − 6 mm) and fixed with four stainless-steel screws on the surface of the skull by dental acrylic resins (Fig. 1C, D) [27]. Nutritional jellies were given for postoperative analgesia (Daily intake: 5 mg/kg, GEL-CPF, Beijing Lab Animal Tech Develop Co., Ltd., Beijing, China), which is a kind of dietary supplement containing carprofen to relieve pain in rodents. Before activating the DBS system, the animals were given a week to recover.

After the seven-day recovery from implantation with the DBS apparatus, the rats underwent behavioral tests to explore any autism-like behavior, spatial learning, and cognitive rigidity. During behavioral testing, animals in the saline-DBS and VPA-DBS groups received high-frequency DBS stimulation (HF-DBS, 120 Hz, 150 µA, 90 µs, rectangular stimulation pulse waveform) continuously for a total of 18 days (Day 11–29). However, neither the saline-sham nor the VPA-sham rats were given any kind of active stimulation throughout the testing period.

## Behavioral tests

The behavioral trace of the rats’ movements in the three-chamber social interaction test and open field test was monitored using smart software (SMART 3.0, Panlab, Spain, supported by RWD Life Science Co., Ltd., China). Before proceeding to the next rat, the equipment was thoroughly disinfected with ethanol at a concentration of 70%. An investigator blinded to all of the groups was responsible for the manual recording of the data for the marble burying test. The data on spatial learning and cognitive rigidity were collected automatically by the IntelliCage system.

## The three-chamber social interaction test

For this research, we utilized a three-chamber version of the social interaction test to assess sociability as well as subjects’ preferences toward social novelty. The protocol was adapted from Crawley [28] and was executed in the manner that had been outlined before [29]. In short, the test was conducted in a rectangular arena (90 cm × 50 cm × 40 cm) composed of three communicating compartments (the left chamber; the right chamber; and the central chamber) divided into equal sections by acrylic glass walls that have apertures in the center to facilitate passage to each chamber. Animals were housed individually and acclimated to the environment for 24 h before testing in the three-chamber device. After being positioned in the middle of the empty apparatus during the habituation phase, the test rat was given ten minutes to freely navigate through each compartment of the device. Then, a novel stranger rat (Stranger 1, S1) was kept under a transparent plastic box in the right chamber (Chamber S1), and the left chamber was left empty (Chamber E). The test animal was kept in the center for another 10 min for free exploration (the sociability phase). Next, another novel stranger rat (Stranger 2, S2) was positioned under a transparent plastic box in the left chamber (Chamber S2), where the S1 rat that was in the sociability phase remained and acted as the familiar animal. After then, the subject animal in the sociability phase was given another ten minutes of unrestricted access to the testing area (the social novelty preference phase). For both the sociability and social novelty preference phases, all behavioral data were analyzed including the time spent in each chamber and the time spent interacting (sniffing) with the novel stranger rat or an empty box. The sociability index, as well as the social novelty preference index, were computed by the differences in the time spent interacting with S1 (or S2) and an empty box (or S1), which were used to assess social motivation or social cognition.

## The open-field test

In this research, the open-field test was used to evaluate locomotor activities, and repetitive/stereotypic-like behaviors [30, 31]. Before the open-field test, the animals were kept in isolation and given twenty-four hours to become accustomed to their new surroundings. The rats being tested were kept inside a box made of dark plastic (49 cm × 49 cm × 30 cm) and the behavioral data were collected for five minutes. The traveled distance, grooming time, and grooming numbers in the test area were analyzed.

## The marble burying test

In our investigation, the marble-burying test was employed to measure repetitive/stereotypic-like behavior, which was adapted as previously described [32]. As used earlier in the open-field test, a standard corncob bedding was placed in a box made of black plastic (49 cm × 49 cm × 30 cm) and filled to a depth of 3 cm. After allowing the test rats to get used to their surroundings for ten minutes, they were returned to their home cage for a short period. During this period, an arrangement of sixteen marbles (1.4 cm marble diameter) in a 4 × 4 pattern was put on the bedding in the box. After that, the test animals were put back in the box and given thirty minutes to bury marbles. A video was made during the experiment to record the behaviors of the rats, and they were photographed after the procedure. A marble that was buried more than 2/3 was considered buried. The final number of buried marbles was recorded and analyzed.

## The intellicage system

Spatial learning and cognitive rigidity were tested with the IntelliCage system, which was carried out in an automated group-housing cage (410 cm × 190 cm × 435 cm). The equipment details of the cage were described as we previously reported and the protocols of the IntelliCage experiment were adapted from a previous study [33–35]. The entire experiment was conducted for a total of 20 days divided into four phases. In Phase 1, rats were mainly acclimated to the new cage for six days. At this stage, rats could access water bottles in all corners ad libitum. The number of cows visits was recorded to assess rat exploratory power and corner preferences. In Phase 2, rats were trained for nose-poke learning within four days, where all doors to the water bottles were closed and the rats had to complete a nose-poke to open the door to access water. The number of times each corner was explored by the rats was counted and recorded to determine the exploratory capacity of the rats as well as their corner preferences for the subsequent tests, especially the least preferred corner. In Phase 3, the cognitive rigidity test was carried out for six consecutive days. In the first three days, During the nose-poke learning stage, the least-preferred corner by the rats was marked as correct, whereas the other corners were marked as “error”. Rats were permitted to go to whatever corner they preferred, but only when the corner was “correct”, could the door be opened, and water allowed For the next three days, the opposite corner of the prior “correct” was labeled as a novel “correct” and the other corners were designated as “error”. Rats were permitted to explore all of the corners listed above. The cognitive rigidity test was measured by determining the ratio of the number of correct corner visits to the total number of all corner visits. In Phase 4, the spatial learning test was conducted within four days. The novel correct corner in Phase 3 with accessible water bottles was rotated clockwise once every day (for four days). As detailed in Phase 3, rats were free to explore all corners. The spatial learning test was measured by computing the ratio of the number of correct corner visits to the total number of all corner visits.

## Verification of electrode position in histology (Nissl staining)

Once all of the behavioral tests had been completed on the rats, they were given a large dose of ketamine (100 mg/Kg) to induce deep anesthesia, and then they were perfused transcardially with 0.9% saline and 4% paraformaldehyde (PFA). Their brains were gently removed and immersed in 4% PFA for eight hours, then submerged in 30% sucrose that dissolved in phosphate-buffered saline (PBS) for 48 h. After being frozen with dry ice, the brains were cut utilizing the HM 430 cryostat microtome (HM 430, Thermo Fisher Scientific, MA, USA) at a thickness of 40 µm. The serial coronal sections were collected at the level of the insula and subsequently stained in 0.1% cresyl violet at 37 ℃ for 15 min. The sections were then rinsed in distilled water and coated with neutral balsam mounting media (G8590, Beijing Solarbio Science & Technology Co., Ltd., Beijing, China). Finally, images were captured using a microscope (DM6, Leica, Germany) to confirm the positioning of the electrode.

## Real-time quantitative PCR (RT-qPCR) analysis

Both the saline-DBS rats and the VPA-DBS rats had DBS electrodes implanted bilaterally in the anterior insula and received continuous stimulation from an electrical current (120 Hz, 150 µA, 90 µs) for a total of 14 days. The animals (3 rats from each saline, VPA, Saline-DBS, and VPA-DBS groups) were deeply anesthetized by isoflurane inhalation (6% induction) and decapitated immediately. After removing the whole brain and mounting it in a brain slice mold that was placed on ice, a slice that was one millimeter in thickness and located proximal to the electrode channel was acquired. After the electrode position was verified, the AI was separated, flash-frozen in liquid nitrogen, and kept at a temperature of – 80 °C. Analyses of the expression of immediate early genes (c-Fos, c-Jun, Arc, and Npas4) were carried out in the AI using RT-qPCR. The total RNA was extracted utilizing a TRIzol reagent (15596018, Thermo Fisher Scientific, MA, USA) following the instructions stipulated by the manufacturer before reverse transcribing them into cDNA utilizing a TRUEscript 1st Strand cDNA Synthesis Kit (PC1803, Aidlab Biotechnologies Co., Ltd., Beijing, China). RT-qPCR was conducted utilizing a SsoAdvanced Universal SYBR Green Supermix kit (1725275, BIO-RAD, California, USA) and 7300 Real-Time PC system (Applied Biosystems, Thermo Fisher Scientific, MA, USA). Analyses were conducted on the expression profiles of c-Fos, c-Jun, Arc, and Npas4 in comparison to the levels of the GAPDH gene transcript.

The following is a list of the primer sequences (5′–3′): c-Fos, forward: GGCTGAACCCTTTGATGA, reverse: GCTGCATAGAAGGAACCA; c-Jun, forward: CATCACCACTACACCGAC, reverse: AGCGTATTCTGGCTATGC; Arc, forward: CCAAACCCAATGTGATCCT, reverse: ACACTTCGGTCAACAGATG; Npas4, forward: AACCCTTCCAGACTCACTT, reverse: TGCTTGGTGTCAACTGTTC; GAPDH, forward: CAGGGCTGCTTTTAACTCTGG, reverse: TGGGTGGAATCATATTGGAACA. The thermal cycling parameters that were employed in the procedure were as follows: initial denaturation for 30 min at 95 °C, 40 cycles at 95 ℃ for 10 s, and 60 °C for 30 s for degeneration and annealing extension. After that, we generated the dissolution curve. The approach known as the 2^−△Cq^ method was employed to compute the relative alterations in gene expression, where Cq represented the quantification cycle.

## Label-free-based proteomics analysis

### Tissue preparation, protein extraction, and digestion

Tissue samples (3 rats from each saline, VPA, and VPA-DBS groups) were collected as described in the RT-qPCR analysis. To lyse the AI in the lysis buffer (8 M urea, 1% protease inhibitor cocktail), a high-intensity ultrasonic processor was utilized to perform three consecutive rounds of sonication on ice. Samples were centrifugated at 12,000 g for ten minutes at a temperature of 4 ℃ to eliminate the residual debris. After collecting the supernatant, a BCA protein quantitative kit was employed to ascertain the concentration of protein present in the sample. Extracted proteins were reduced with five mM dithiothreitol for 30 min at 56 °C before alkylation with 11 mM iodoacetamide in darkness at ambient temperature for 15 min. After that, the content of the protein samples was diluted by attaining a certain urea solution (< 2 M concentration) with 100 mM NH4HCO3. During the first digestion, which took place throughout the night, trypsin was introduced at a ratio of 1:50 trypsin to protein mass. During the second digestion, which took place for four hours, the trypsin was introduced at a ratio of 1:100 trypsin to protein mass.

## LC–MS/MS analysis

After being dissolved in solvent A (0.1 percent formic acid), the tryptic peptides were directly loaded onto a reversed-phase analytical column that measured 15 cm in length and had an internal diameter of 75 μm. The gradient consisted of an increase in solvent B (0.1% formic acid in 98% acetonitrile) from 6 to 23% over 26 min, from 23 to 35% in eight minutes, from 35 to 80% in 3 min, and then remaining at 80% for the remaining 3 min, while all maintaining a flow rate of 400 ml/min across the EASY-nLC 1000 UPLC system. After going through an NSI source, the peptides were subjected to a tandem mass spectrometry (MS/MS) analysis using a Q Exactive Plus (Thermo Fisher Scientific, MA, USA) that was connected online to the UPLC. During the electrospray process, a voltage of 2.0 kV was utilized. The scan had an m/z range of 350 to 1800, and at 70,000 resolution, complete peptides were identified in the Orbitrap. After that, peptides were subjected to MS/MS analysis by setting the NCE to a value of 28, and the fragments were recognized by the Orbitrap operating at 17,500 resolution. A data-dependent process that alternated between one MS scan followed by 20 MS/MS scans with 15.0 s dynamic exclusion was performed. The automatic gain control was calibrated to a value of 5E4.

## Database search

The identification of the generated MS/MS data was performed with the PEAKS Studio (Bioinformatics Solutions, Ontario, CA). The Uniprot Rattus norvegicus database was integrated with the reverse decoy database, and then tandem mass spectra were screened against both of those databases. Trypsin/P served as the cleavage enzyme because it allows for up to two cleavages to be omitted. In the initial inquiry, the mass tolerance for precursor ions was adjusted at 20 ppm, and in the subsequent primary inquiry, it was set at 5 ppm. In addition, a mass tolerance of 0.02 Da was chosen for fragment ions. The carbamidomethyl on Cys was stated as the constant modification, whereas the oxidation on Met was set as the variable modification. The LFQ technique was utilized for label-free quantification; the FDR was established at  < 1%, and the required minimum score for peptides was established at  > 40.

## Bioinformatics analysis

All of the samples that came from a single treatment batch were integrated into a single pool, and the duplicates that were performed were technical copies of the pooled sample. To detect the differential expression proteins (DEPs), a calculation was made to determine the fold-change (ratio < 0.67 or > 1.5, P < 0.05) of the proteins. The comparison group (Saline vs. VPA) was performed in this study, the GO analysis was performed to assign the DEPs into the biological process (BP), molecular functions (MF), and cellular components (CC) ontology with the help of the InterProScan tool. The KEGG database, together with an automated annotation server (KAAS), was utilized to provide a pathway description for the DEPs that were identified. The bottleneck algorithm of Cytohubba software was utilized in the analysis of hub proteins and protein-protein interaction (PPI) network. The fuzzy c-means algorithm is a new clustering algorithm. To eliminate the proteins with substantial changes in protein abundance in the saline, the VPA, and VPA-DBS samples, the relative expression level of the protein was first converted by the Log2 logarithm before screening out the proteins whose SD > 0. The selected proteins were employed for the expression profile cluster analysis of the Mfuzz method. To thoroughly elucidate the biological processes involved in the proteins in each cluster, we separately analyzed the Gene Ontology (GO) function and the Kyoto Encyclopedia of Genes and Genomes (KEGG) pathway of the proteins in each cluster.

## Statistical analysis

Data were displayed as mean  ± standard deviation (SD). A two-way repeated-measures analysis of variance (ANOVA) was implemented to determine statistical variations in the behavioral tests before DBS was turned off, and posthoc analyses were completed utilizing the Bonferroni multiple comparisons test. To analyze the data of behavioral tests after DBS was turned off, the IntelliCage system, and qPCR results, a one-way ANOVA was employed to determine statistical variations, and posthoc analyses were performed using Tukey’s multiple comparisons test. *P*-value < 0.05 was established as the criterion for statistical significance. R version 3.6.3 and GraphPad Prism 7 (GraphPad Software, La Jolla, CA) were employed to execute all analyses of statistical data and generate graphs, correspondingly.

## Results

### Verification of the DBS electrode target

After completing all of the behavioral tests on the animals, they were subsequently sacrificed, and the electrodes were removed thereafter. In addition to this, we made brain serial coronal slices that had been dyed with cresyl violet and confirmed the placement of the electrodes. In the examination of the data, only rats that had the electrodes in the AI installed properly were considered valid. Figure 1C, D illustrate the positions of the electrode tips located in the AI of several groups of animals.

## HF-DBS reversed autism-like behavior in VPA-exposed rats

The autism-like behavioral tests were conducted on days 7, 13, 18, 25, and 36. In contrast, two-way repeated-measures ANOVA tests illustrated there was no considerable alteration in autism-like behavior among the saline and saline-sham groups or the VPA and VPA-sham groups on days 7, 13, 18, and 25 (Additional file 1: Figure S1A–G, Additional file 2: Figure S2A–E). This indicates that animal behavior was not affected by the way of electrode implantation.

**Fig. 2:**
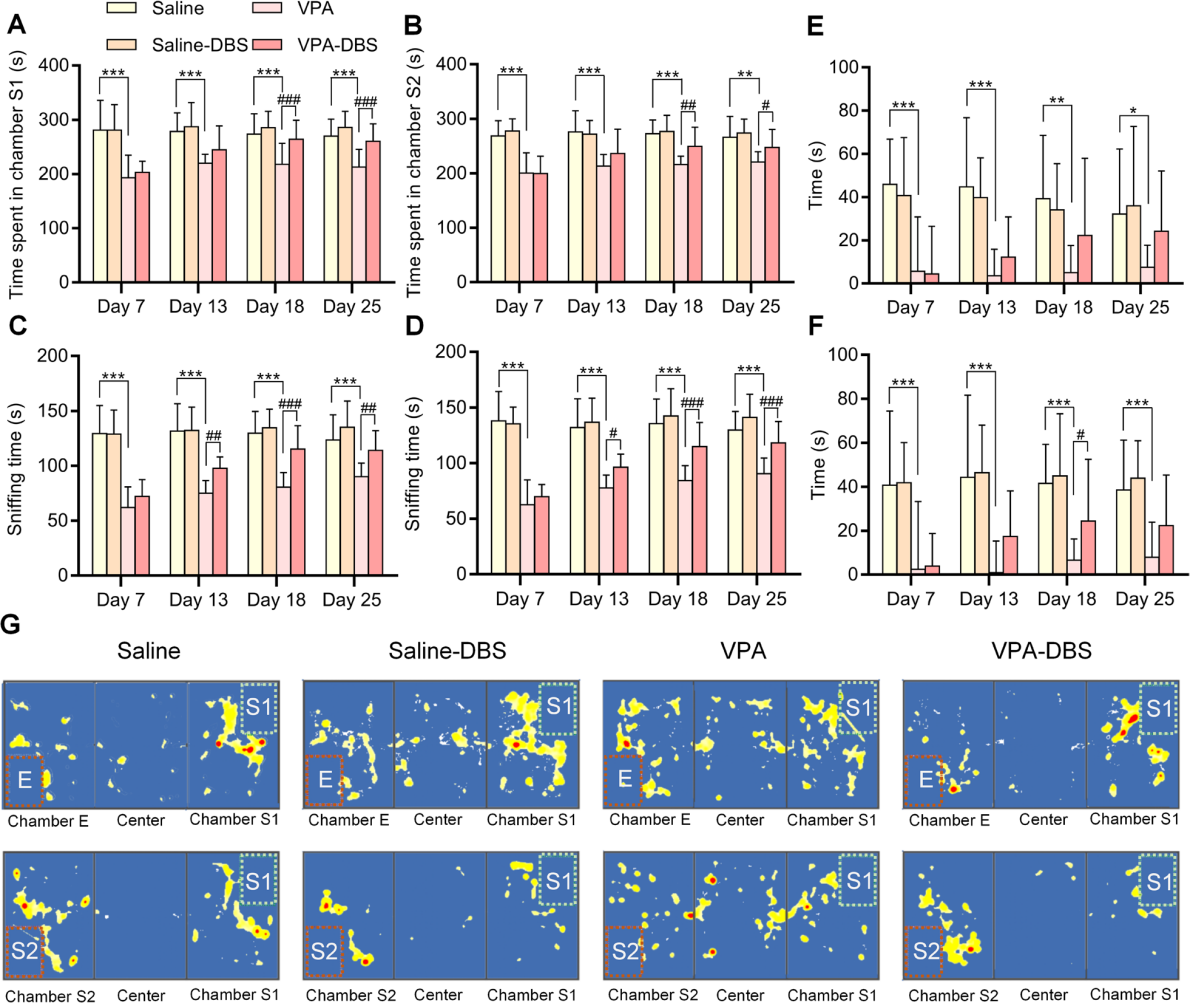
HF-DBS improved the social ability in the VPA-exposed offspring. In three-chamber social interaction test: **A** Time spent by test rats in chamber S1. **B** Time spent by test rats in chamber S2. **C** Sniffing time between test rats and S1. **D** Sniffing time between test rats and S2. **E** Sociability index. **F** Social novelty preference index. **G** Representative heat maps of three-chamber social interaction test on day 18. Data are shown as mean with SD. Two-way repeated ANOVA with post-hoc Bonferroni test, ^*^p < 0.05 vs. saline; ^**^p < 0.01 vs. saline; ^***^p < 0.001 vs. saline; ^#^*p* < 0.05 vs. VPA; ^##^*p* < 0.01 vs. VPA; ^###^*p* < 0.001 vs. VPA

**Fig. 3:**
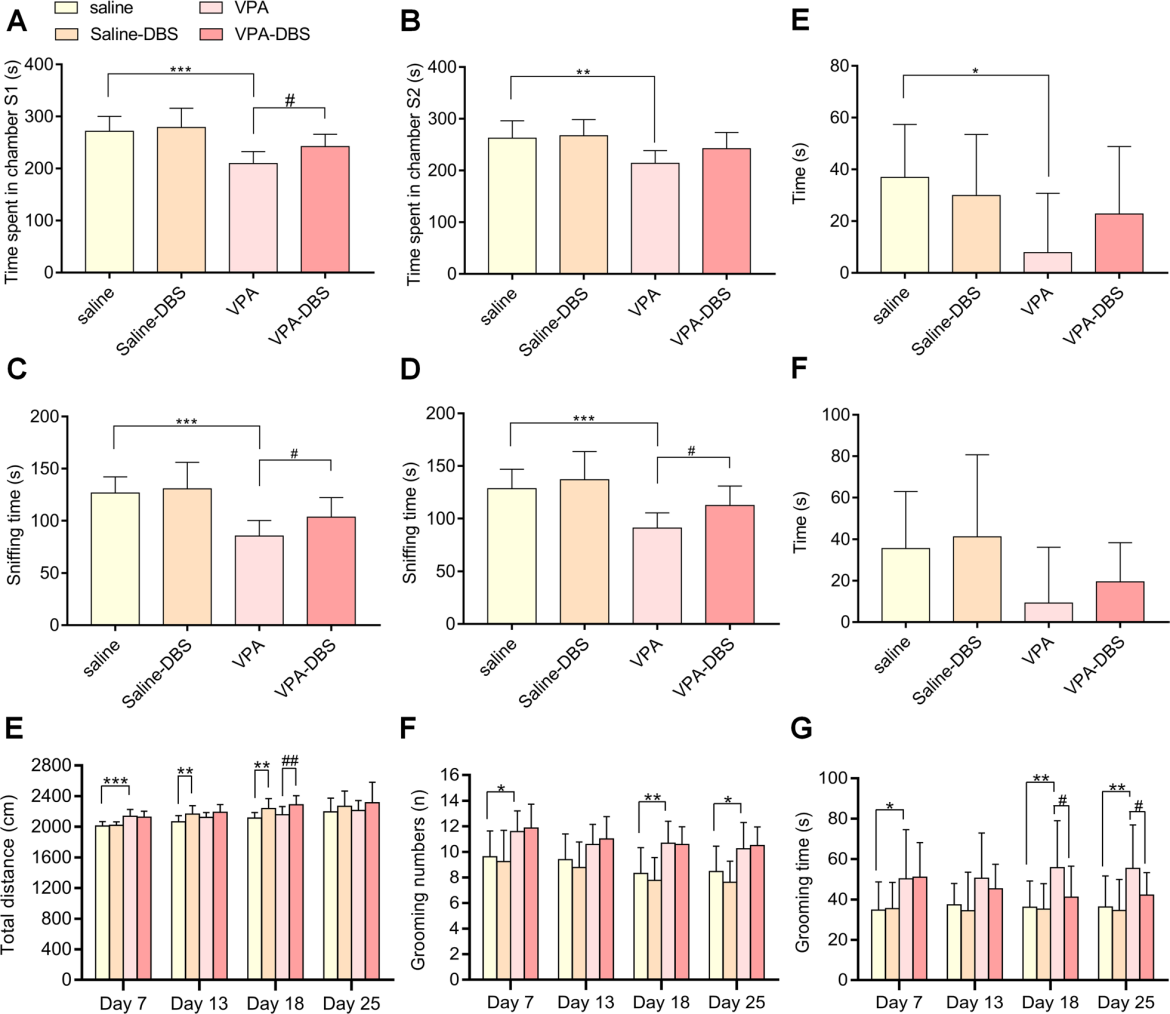
When HF-DBS was turned off for 7 days (Day 36), HF-DBS still reversed the decreased social interaction. **A** Time spent by test rats in Chamber S1 on day 36. **B** Time spent by test rats in Chamber S2 on day 36. **C** Sniffing time between test rats and S1 on day 36. **D** Sniffing time between test rats and S2 on day 36. **E** Sociability index on day 36. **F** Social novelty preference index on day 36. Data are shown as mean with SD. One-way ANOVA with post-hoc Tukey's test, ^*^p < 0.05 vs. saline; ^**^p < 0.01 vs. saline; ^***^p < 0.001 vs. saline; ^#^*p* < 0.05 vs. VPA. HF-DBS alleviated the excessive repetitive behavior in the VPA-exposed offspring. **G** Total distance travelled by test rats. **H** Number of self-grooming behavior. **I** Duration of self-grooming behavior. Two-way repeated ANOVA with post-hoc Bonferroni test, ^*^p < 0.05 vs. saline; ^**^p < 0.01 vs. saline; ^***^p < 0.001 vs. saline; ^#^*p* < 0.05 vs. VPA; ^##^*p* < 0.01 vs. VPA; ^###^*p* < 0.001 vs. VPA

In the three-chamber social interaction experiment, we discovered that the VPA-exposed rats spent significantly shorter time in Chamber S1 or Chamber S2 (the repeated measurements using a two-way ANOVA showed that the amount of time spent in Chamber S1 on days 7, 13, 18, and 25 was *p* < 0.001; the time spent in Chamber S2 on days 7, 13, and 18 was *p* < 0.001, on day 25 was *p* < 0.01), and the HF-DBS contributed to a considerable increase in the time spent in Chamber S1 and Chamber S2 in the VPA-DBS group in contrast with the VPA group (The two-way repeated measures ANOVA illustrated the time spent in Chamber S1 on day 7 was *p* = 0.561, on day 13 with *p* = 0.083, days 18 and 25 with *p* < 0.001, with Group: *F*_(5, 71)_ = 37.101, *p* < 0.001; Time: *F*_(3, 213)_ = 3.489, *p* < 0.05; and Interaction: *F*_(15, 213)_ = 1.662, *p* = 0.060. The repeated measurements using a two-way ANOVA illustrated the time spent in Chamber S2 on day 7 was *p* = 0.953, on day 13 with *p* = 0.075, day 18 with *p* < 0.01, and day 25 with *p* < 0.05, with Group: *F*_(5, 71)_ = 31.578, *p* < 0.001; Time: *F*_(3, 213)_ = 3.335, *p* < 0.05; and Interaction: F_(15, 213)_ = 1.359, *p* = 0.170) (Fig. 2A, B). The rats that were given VPA spent a much shorter amount of time interacting with either S1 or S2 (Repeated measurements using a two-way ANOVA illustrated that the sniffing time spent with S1 on days 7, 13, 18, and 25 at *p* < 0.001; and sniffing time spent with S2 on days 7, 13, 18, and 25 was *p* < 0.001). The decreased sniffing time spent with S1 or S2 were reversed by HF-DBS in the VPA-DBS group (Repeated measurements using a two-way ANOVA illustrated that the sniffing time spent with S1 on day 7 was *p* = 0.219, days 13 and 25 was *p* < 0.01, and day 18 was *p* < 0.001, with Group: *F*_(5, 71)_ = 87.776, *p* < 0.001; Time: *F*_(3, 213)_ = 10.488, *p* < 0.001; Interaction: *F*_(15, 213)_ = 3.078, *p* < 0.001. As per the findings of repeated measures of two-way ANOVA, the sniffing time spent with S2 on day 7 was *p* = 0.346, day 13 was *p* < 0.05, and days 18 and 25 was *p* < 0.001, with Group: *F*_(5, 71)_ = 84.107, *p* < 0.001; Time: *F*_(3, 213)_ = 16.661, *p* < 0.001; Interaction: *F*_(15, 213)_ = 3.942, *p* < 0.001) (Fig. 2C, D). The social novelty preference and sociability indices in the VPA-exposed rats were substantially reduced (Repeated measurements using a two-way ANOVA illustrated that the sociability index for days 7 and 13 was *p* < 0.001, day 18 was *p* < 0.01, and day 25 was *p* < 0.05; and the social novelty preference index on days 7, 13, 18, and 25 was *p* < 0.001). The decreased sociability and social novelty preference indices were reversed in the VPA-DBS group, but with the exception of day 18, there was no substantial difference in contrast with the VPA group (The two-way repeated measures ANOVA revealed the sociability index on day 7 was *p* = 0.898, day 13 was *p* = 0.293, day 18 was *p* = 0.080, and day 25 was *p* = 0.104, with Group: *F*_(5, 71)_ = 20.964, *p* < 0.001; Time: *F*_(3, 213)_ = 0.093, *p* = 0.964; and Interaction: *F*_(15, 213)_ = 0.910, *p* = 0.554. The repeated measurements using a two-way ANOVA illustrated that the social novelty preference index on day 7 was *p* = 0.881, day 13 was *p* = 0.072, day 18 was *p* < 0.05, and day 25 was *p* = 0.059, with Group: *F*_(5, 71)_ = 25.340, *p* < 0.001; Time: *F*_(3, 213)_ = 0.949, *p* = 0.418; and Interaction: *F*_(15, 213)_ = 0.575, *p* = 0.892) (Fig. 2E, F). The representative heat maps of the three-chamber social interaction experiment on day 25 is displayed in Fig. 2G. When DBS systems were turned off for 7 days (On day 36), one-way ANOVA showed that there was a significant change in the time spent in Chamber S1 or Chamber S2 among the saline and VPA groups (Time spent in Chamber S1: *F*_(3,48)_ = 15.29, *p* < 0.001; Time spent in Chamber S2: *F*_(3,48)_ = 7.762, *p* < 0.01), and the effect of HF-DBS may persisted (Time spent in Chamber S1: *F*_(3,48)_ = 15.29, *p* < 0.05), but there was no significant change in time spent in Chamber S2 among VPA and VPA-DBS group (Time spent in Chamber S2: *F*_(3,48)_ = 7.762, *p* = 0.091) (Fig. 3A, B)**.** The one-way ANOVA showed that there was a significant change in the sniffing time spent with S1 or S2 among the saline and VPA groups (Sniffing time spent with S1: *F*_(3,48)_ = 15.01, *p* < 0.001; Sniffing time spent with S2: *F*_(3,48)_ = 12.32, *p* < 0.001), and the effect of HF-DBS persisted (Time spent in Chamber S1: *F*_(3,48)_ = 15.01, *p* < 0.01; Time spent in Chamber S2: *F*_(3,48)_ = 12.32, *p* < 0.01) (Fig. 3C, D). Futhermore, one-way ANOVA showed that there was a significant change in the sociability index among the saline and VPA groups (*F*_(3,48)_ = 3.503, *p* < 0.05), and social novelty preference index was decreased in VPA groups (*F*_(3,48)_ = 3.233, *p* = 0.131). The effect of HF-DBS may persisted, but there was no significant change (Sociability index: *F*_(3,48)_ = 3.503, *p* = 0.376; Social novelty preference index: *F*_(3,48)_ = 3.233, *p* = 0.605) (Fig. 3E, F). The above results indicate that the social ability of VPA-exposed rats was severely impaired. HF-DBS reversed cognitive social barriers to the greatest extent on day 18, and this effect persisted after stimulation ceased. In addition, this treatment had no obvious effect on the social abilities of the saline-treated rats.

**Fig. 4:**
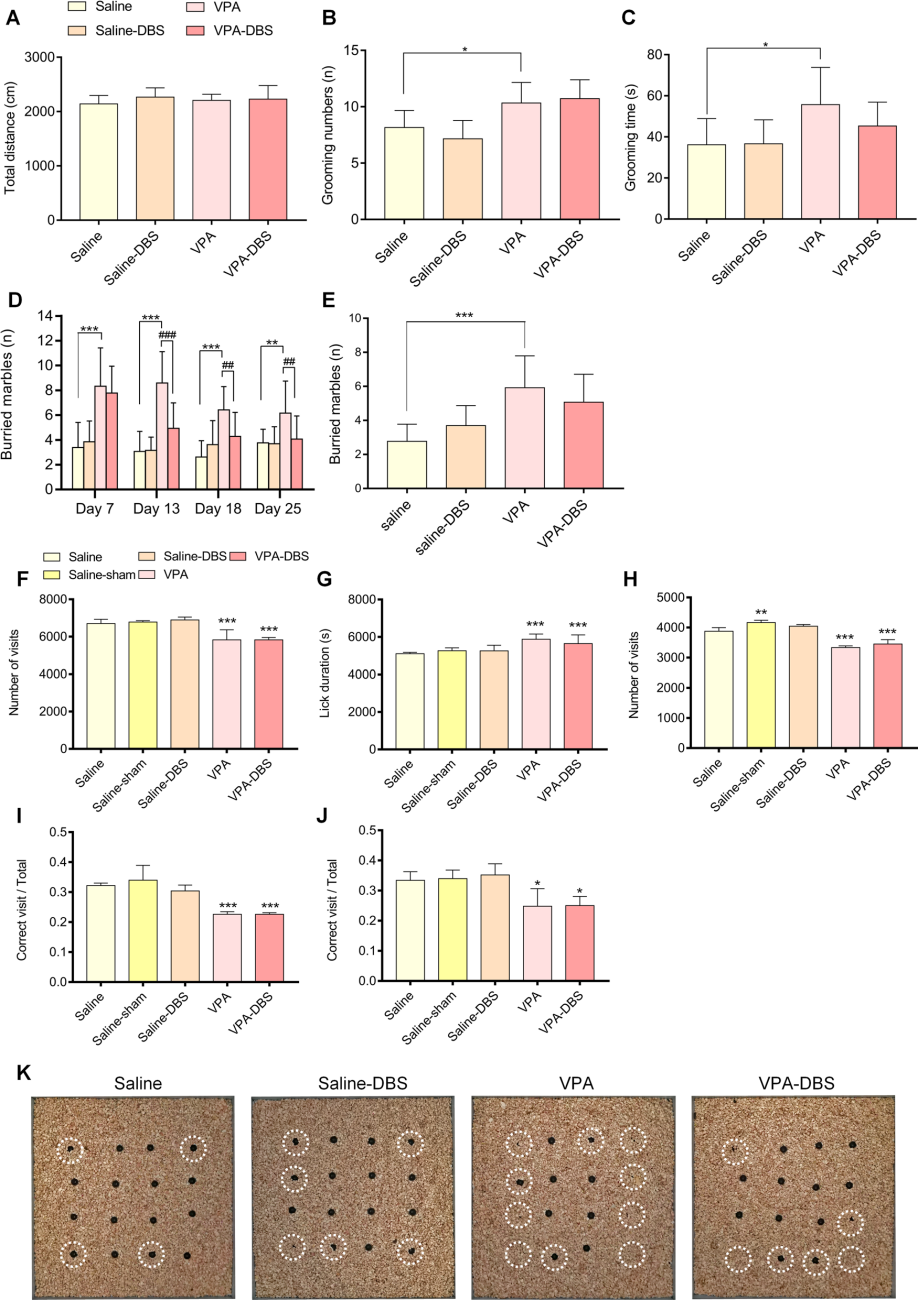
When HF-DBS was turned off for 7 days (Day 36), the effect of HF-DBS on repetitive behavior was weakened. **A** Total distance travelled by test rat on day 36. **B** Number of self-grooming behavior on day 36. **C** Duration of self-grooming behavior on day 36. One-way ANOVA with post-hoc Tukey’s test, ^*^p < 0.05 vs. saline; ^#^*p* < 0.05 vs. VPA. HF-DBS reversed the repetitive/stereotypic-like activities in marble burying test: **D** Number of marbles buried. Data are shown as mean with SD. Two-way repeated ANOVA with post-hoc Bonferroni test, ^**^p < 0.01 vs. saline; ^***^p < 0.001 vs. saline; ^##^*p* < 0.01 vs. VPA; ^###^*p* < 0.001 vs. VPA. **E** Number of marbles buried on day 36. Data are shown as mean with SD. one-way ANOVA with post-hoc Tukey's test, ^***^p < 0.001 vs. Saline. There is no significant effect on spatial learning ability and cognitive rigidity in the VPA-exposed offspring. **F** Number of total corner visits in phase 1. **G** Lick dutation of all corners in phase 1. **H** Number of total corner visits in phase 2. **I** Ratio of the number of correct corner visits to the number of total corner visits in phase 3. **J** Ratio of the number of correct corner visits to the number of total corner visits in phase 4. Data are shown as mean with SD. Ono-way ANOVA with post-hoc Tukey's test, ^*^ *p* < 0.05 vs. saline; ^**^ *p* < 0.01 vs. saline; ^***^ *p* < 0.001 vs. saline. **K** Representative marbles buried maps of marbel burying test on day 18

The findings of the open-field test showed that HF-DBS was the cause of a slight increase in the total distance covered by rats in the DBS groups (The repeated measurements with a two-way ANOVA illustrated that the total distance covered by the saline-DBS group on day 7 was *p* = 0.804, day 13 was *p* < 0.01, day 18 was *p* < 0.01, and day 25 was *p* = 0.742, while the VPA-DBS group on day 7 was *p* = 0.669, day 13 was *p* = 0.062, day 18 was *p* < 0.01, and day 25 was *p* = 0.878 with Group: *F*_(5, 71)_ = 9.309, *p* < 0.001; Time: *F*_(3, 213)_ = 18.870, *p* < 0.001; and Interaction: *F*_(15, 213)_ = 1.352, *p* = 0.205) (Fig. 3E). The grooming numbers and grooming time of the VPA group were substantially elevated (The two-way repeated measures ANOVA illustrated the grooming numbers on days 7 and 25 was *p* < 0.05, day 13 was *p* = 0.130, and day 18 was *p* < 0.01 while the grooming time on day 7 was *p* < 0.05, day 13 was p = 0.057, and days 18 and 25 was *p* < 0.01), and the increased grooming time was reversed in the VPA-DBS group, but there was no remarkable change in grooming numbers in the VPA-DBS group (The repeated measurements with a two-way ANOVA illustrated that the grooming numbers on day 7 was *p* = 0.723, day 13 was *p* = 0.590, day 18 was *p* = 0.891, and day 25 was *p* = 0.729, with Group: *F*_(5, 71)_ = 25.918, *p* < 0.001; Time: *F*_(3, 213)_ = 7.895, *p* < 0.001; and Interaction: *F*_(15, 213)_ = 0.152, *p* > 0.999. The repeated measurements with a two-way ANOVA illustrated that the grooming time on day 7 was *p* = 0.890, day 13 was *p* = 0.436, day 18 was *p* < 0.05, and day 25 was *p* < 0.05, with Group: *F*_(5, 71)_ = 10.492, *p* < 0.001; Time: *F*_(3, 213)_ = 0.025, *p* = 0.994; and Interaction: *F*_(15, 213)_ = 0.370, *p* = 0.985) (Fig. 3F, G). On day 36, there was no significant difference in the total distance travelled among saline and saline-DBS or VPA and VPA-DBS groups (One-way ANOVA, *F*_(3,48)_ = 1.073, saline-DBS: *p* = 0.316; VPA-DBS: *p* = 0.986), and total distance in VPA groups were not significantly different compared with saline group (One-way ANOVA, *F*_(3,48)_ = 1.073, *p* = 0.821) (Fig. 4A). In addition, the one-way ANOVA demonstrated a remarkable change in grooming numbers and grooming time among the saline and VPA groups (One-way ANOVA, grooming numbers: *F*_(3,48)_ = 13.92, *p* < 0.05; grooming time: *F*_(3,48)_ = 5.597, *p* < 0.05), and decreased grooming time may be persisted, but there was no significance (One-way ANOVA, grooming time: *F*_(3,48)_ = 5.597, saline-sham: *p* > 0.999; VPA-sham: *p* = 0.232) (Fig. 4B, C). As mentioned above, the excessive repetitive behaviors were observed in VPA-exposed rats. HF-DBS appeared to reduced excessive grooming time on day 13. Other than the possibility of this intervention increasing activity in the rats, this treatment had no other significant effect on behavior in the saline-treated rats in the open-field experiment.

For the marble burying test, the results highlighted that the VPA-exposed rats buried significantly more marbles (the repeated measurements with a two-way ANOVA illustrated that the number of marbles buried on days 7, 13, and 18 was *p* < 0.001, and day 25 was *p* < 0.01), and HF-DBS contributed to a remarkable reduction in the number of marbles buried in the VPA-DBS subgroup (The repeated measurements with a two-way ANOVA illustrated that the number of marbles buried on day 7 was *p* = 0.517, day 13 was *p* < 0.001, and days 18 and 25 was *p* < 0.01, with Group: *F*_(5, 71)_ = 43.222, *p* < 0.001; Time: *F*_(3, 213)_ = 8.526, *p* < 0.001; and Interaction: *F*_(15, 213)_ = 2.680, *p* < 0.001) (Fig. 4D). On day 36, the one-way ANOVA highlighted that there was a considerable change in the number of marbles buried among the saline and VPA groups (the one-way ANOVA, *F*_(3,48)_ = 11.64, *p* < 0.001), and the number of marbles buried in the VPA-DBS group decreased, however, there was no significance (The one-way ANOVA indicating *F*_(3,48)_ = 11.64, *p* = 0.465) (Fig. 4E). The representative marble buried maps of the marble burying test on day 25 are exhibited in Fig. 4K. The repetitive/stereotypic-like manifestations of the VPA-exposed rats were observed in this test. HF-DBS appeared to reverse the behavior on day 13, and this effect persisted in subsequent treatments of stimulation. Adverse effects caused by HF-DBS were not observed in the saline-exposed rats.

## HF-DBS does not influence the spatial learning and rigidity in VPA-exposed rats

Using the IntelliCage system, we conducted further exploration to determine whether or not continuous HF-DBS affected learning, memory, and cognitive rigidity. In phase 1, rats were used to assess exploratory power and corner preferences. The results revealed that the number of corner visits in the VPA and VPA-DBS groups decreased (The one-way ANOVA revealed *F*_(4, 20)_ = 6.212, and for the VPA group, it was *p* < 0.01, the VPA-DBS group was *p* < 0.01) (Fig. 4F), and the licking duration increased (The one-way ANOVA was *F*_(4, 20)_ = 18.33, with the VPA group at *p* < 0.001, and the VPA-DBS group with *p* = 0.240) (Fig. 4G). No remarkable alteration in the number of corner visits and licking duration among the saline, saline-sham, and saline-DBS groups (The one-way ANOVA revealed the number of corner visits in the saline-sham group was *p* = 0.992, and in the saline-DBS subgroup was *p* = 0.810. The one-way ANOVA revealed the licking duration of the saline-sham group was *p* = 0.903, and the saline-DBS group was *p* = 0.919) (Fig. 4F, G). HF-DBS did not affect the number of corner visits and licking duration in the VPA-DBS group (The one-way ANOVA revealed the number of corner visits was *p* > 0.999 and licking duration was *p* = 0.708) (Fig. 4F, G). In Phase 2, the rat exploratory power and corner preferences were analyzed, particularly the least-preferred corner. The number of corner visits in the VPA and VPA-DBS groups decreased (The one-way ANOVA revealed *F*_(4, 20)_ = 68.01, the VPA group was *p* < 0.001, VPA-DBS group was *p* < 0.001), and HF-DBS did not affect the number of corner visits in the VPA-DBS group (The one-way ANOVA revealed the number of corner visits was *p* = 0.354) (Fig. 4H). The number of corner visits in the Saline-sham group increased (The one-way ANOVA revealed that *F*_(4, 20)_ = 68.01, *p* < 0.01) (Fig. 4H). In Phase 3, cognitive rigidity was analyzed in this stage. The proportion of the number of correct corner visits to the total corner visits was reduced in the VPA and VPA-DBS groups (The one-way ANOVA revealed *F*_(4, 20)_ = 24.31, and the VPA group was *p* < 0.001, the VPA-DBS group was *p* < 0.001), and HF-DBS did not affect the ratio in the VPA-DBS group (The one-way ANOVA illustrated *p* > 0.999) (Fig. 4I). No substantial change was identified in the proportion among the saline, saline-sham, and saline-DBS groups (The one-way ANOVA indicated the saline-sham group was *p* = 0.772, and the saline-DBS group was *p* = 0.773) (Fig. 4I). In Phase 4, the spatial learning ability was analyzed in this stage. The proportion of the number of correct corner visits to the total corner visits reduced in the VPA and VPA-DBS groups (The one-way ANOVA highlighted that *F*_(4, 20)_ = 8.946, the VPA group was *p* < 0.05, and VPA-DBS group was *p* < 0.05), and HF-DBS did not affect the ratio in the VPA-DBS group (The one-way ANOVA was *p* > 0.999) (Fig. 4J**)**. No remarkable change was found in the ratio among the saline, saline-sham, and saline-DBS groups (The one-way ANOVA, for the saline-sham group, was p = 0.999, and for the saline-DBS group was *p* = 0.944) (Fig. 4J). The above results indicate that the spatial learning abilities of the VPA-exposed rats were impaired and accompanied by cognitive rigidity. Continuous HF-DBS appeared to not damage the spatial learning abilities of the saline-exposed rats. Unfortunately, the cognitive impairment in the VPA rats cannot be reversed.

## The expression of immediate early genes (IEGs)

**Fig. 5:**
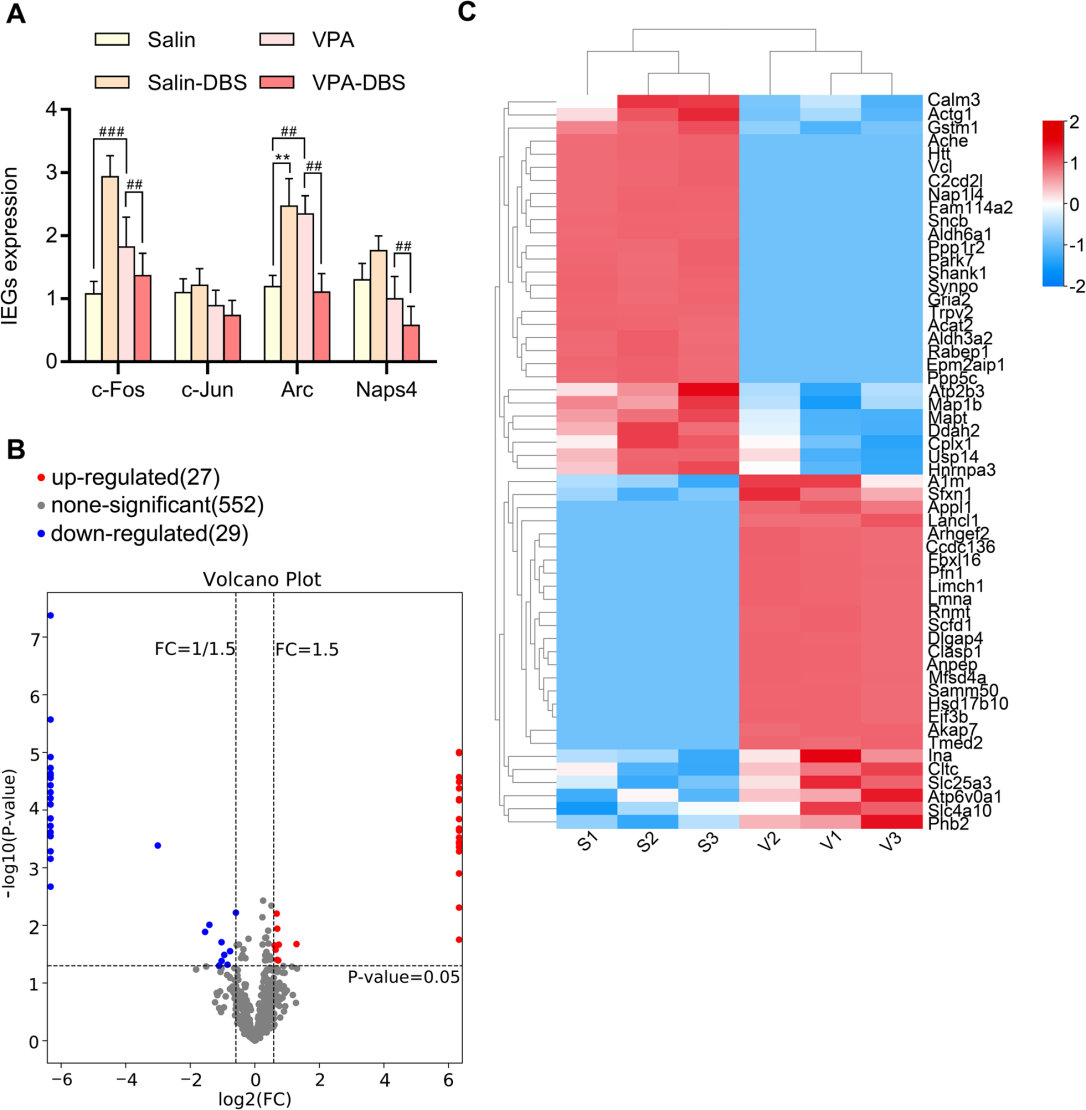
**A** Expression levels of immediate early genes in the insular cortex, including c-Fos, c-Jun, Arc, Naps4. ^**^p < 0.01 vs. saline; ^##^*p* < 0.01 vs. VPA; ^###^*p* < 0.001 vs. VPA. **B** Volcano plot demonstrating proteins differentially regulated in VPA compared to saline. Each data point represents a single quantified protein. Proteins in blue indicate for downregulation and in red indicate for upregulation in VPA. The abscissa of the volcano graph is log2 (FC), the farther the value from zero, the greater the difference, with upregulation on the right and downregulation on the left. The ordinate is -log10 (P-value), and the farther the ordinate value from zero, the greater the difference. **C** Heat map showing the up- and down-regulated proteins with a *p* value  < 0.05 and  ≥ 1.5-fold change between saline and VPA

To examine if the HF-DBS protocol could regulate the activity in the AI after 14 days of electrode stimulation, we further analyzed the immediate early genes expression (c-Jun, Arc, c-Fos, and Npas4) in the AI (Fig. 5A). In contrast with the rats given saline as a control, the number of c-Fos and Arc expressed by the VPA animals was much higher (The one-way ANOVA highlighted that c-Fos was *F*_(3,8)_ = 15.898, *P* = 0.001; and Arc was *F*_(3,8)_ = 16.105, *P* < 0.01). Additionally, the levels of Npas4 Arc, and c-Fos, but not c-Jun transcripts were remarkably decreased in the VPA-DBS rats as opposed to the VPA rats (The one-way ANOVA illustrated c-Fos: *F*_(3,8)_ = 15.898, *P* < 0.01; Arc: *F*_(3,8)_ = 16.105, *P* < 0.01; Npas4: *F*_(3,8)_ = 8.769, *P* < 0.01; and c-Jun: *F*_(3,8)_ = 2.341, *P* = 0.149). Surprisingly, in contrast with the levels found in the saline rats, the levels of c-Fos and Arc found in the saline-DBS animals were considerably higher (The one-way ANOVA indicating c-Fos: *F*_(3,8)_ = 15.898, *P* = 0.123; and Arc: *F*_(3,8)_ = 16.105, *P* < 0.01). Thus, these findings indicate that HF-DBS could change the activity state in the AI.

## MS-based quantitative proteomics between VPA and saline batches

**Fig. 6:**
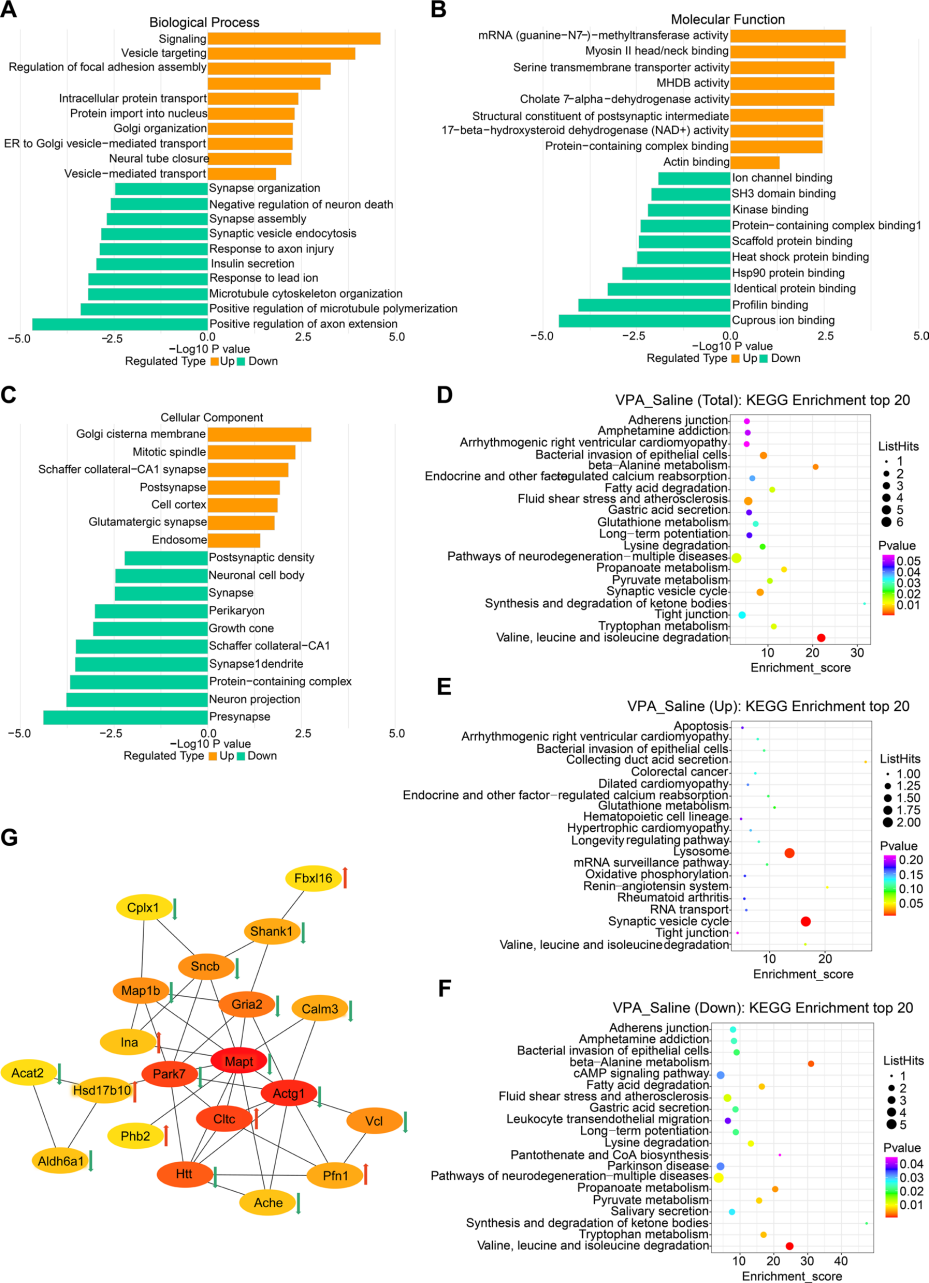
**A** GO analysis of the significant proteins on their biological process, **B** molecular function and **C** cellular component. Yellow indicates the up-regulated and green the down-regulated terms. Bubble chart of the KEGG enrichment analysis. The x-axis is the enrichment score and the y-axis is the pathway terms. The size of the bubbles reflects the number of differentially expressed proteins. The larger the bubble, the greater the number of differentially expressed proteins. The lower the P-value, the higher the significance of the KEGG pathway enrichment. **D** Enrichment pathways screened among total differentially expressed proteins. **E** Enriched pathways screened among up-regulated proteins. **F** Enriched pathways screened among down-regulated proteins. **G** PPI of 20 hub proteins. In hub protein analysis, the darker the color, the higher the score. *KEGG* Kyoto Encyclopedia of Genes and Genomes, *GO* Gene Ontology, *PPI* protein-protein interaction

The quantitative proteome methodology premised on label-free coupled with tandem mass spectrometry (LC–MS/MS) was utilized to examine the differences in the AI’s proteomic profile that were observed between the VPA and saline groups. In total, we identified up to 2939 proteins, of which 1872 proteins were identified in all samples. Fifty-six DEPs, including 27 up-modulated and 29 down-modulated, were identified in the VPA vs. saline batches (Fig. 5B–C, Additional file 3: Table S1). Gene Ontology (GO) enrichment analyses were performed concerning biological processes (BP), cellular components (CC), and molecular functions (MF). As for BP, the up-regulated DEPs were remarkably enriched in signaling, vesicle targeting, the modulation of focal adhesion assembly, the positive modulation of epithelial cell migration, intracellular protein transport, the import of protein into the nucleus, Golgi organization, ER to Golgi vesicle-regulated transport, neural tube closure, vesicle-mediated transport, and the down-regulated DEPs were all significantly enriched in the synapse organization, negative regulation of neuron death, synapse assembly, synaptic vesicle endocytosis, response to axon injury, the positive regulation of axon extensions, insulin secretion, response to lead ion, microtubule the positive modulation of microtubule polymerization, and cytoskeleton organization (Fig. 6A). For CC, the up-regulated DEPs were significantly enriched in the Golgi cisterna membrane, mitotic spindle, Schaffer collateral-CA1 synapse, postsynapse, cell cortex, glutamatergic synapse, endosome, and the down-regulated DEPs were significantly enriched in the postsynaptic density, neuronal cell body, synapse, perikaryon, growth cone, Schaffer collateral-CA1 synapse 1, dendrite, protein-containing complex, neuron projection, and presynapse (Fig. 6B). As for MF, the up-regulated DEPs significantly enriched in mRNA (guanine-N7-)-methyltransferase activity, myosin II head/neck binding, D − serine transmembrane transporter activity, MHDB activity, cholate 7-alpha-dehydrogenase activity, structural constituent of postsynaptic intermediate, 17-beta-hydroxysteroid dehydrogenase (NAD +) activity, protein-containing complex binding, actin binding, and the down-modulated DEPs were significantly enriched in ion channel binding, identical protein binding, SH3 domain binding, Hsp90 protein binding, kinase binding, scaffold protein binding, protein-containing complex binding1, heat shock protein binding, profilin binding, and cuprous ion binding (Fig. 6C).

To comprehend the biological activities, reaction networks, and particular pathways linked to DEPs in both the saline and the VPA batches, a KEGG pathway analysis was conducted. The topmost 20 KEGG pathways in the VPA vs. saline batches were closely related to Valine, leucine, and isoleucine degradation, neurodegeneration-multiple diseases, synaptic vesicle cycle, fluid shear stress and atherosclerosis, bacterial invasion of epithelial cells, and tight junction (Fig. 6D). As for up-regulated DEPs, the most relevant pathways included synaptic vesicle cycles and lysosomes (Fig. 6E). The closely related pathways of down-regulated DEPs included neurodegeneration-multiple diseases, fluid shear stress and atherosclerosis, Parkinson's disease, cAMP signaling pathway, and Valine, leucine, and isoleucine degradation (Fig. 6F).

**Fig. 7:**
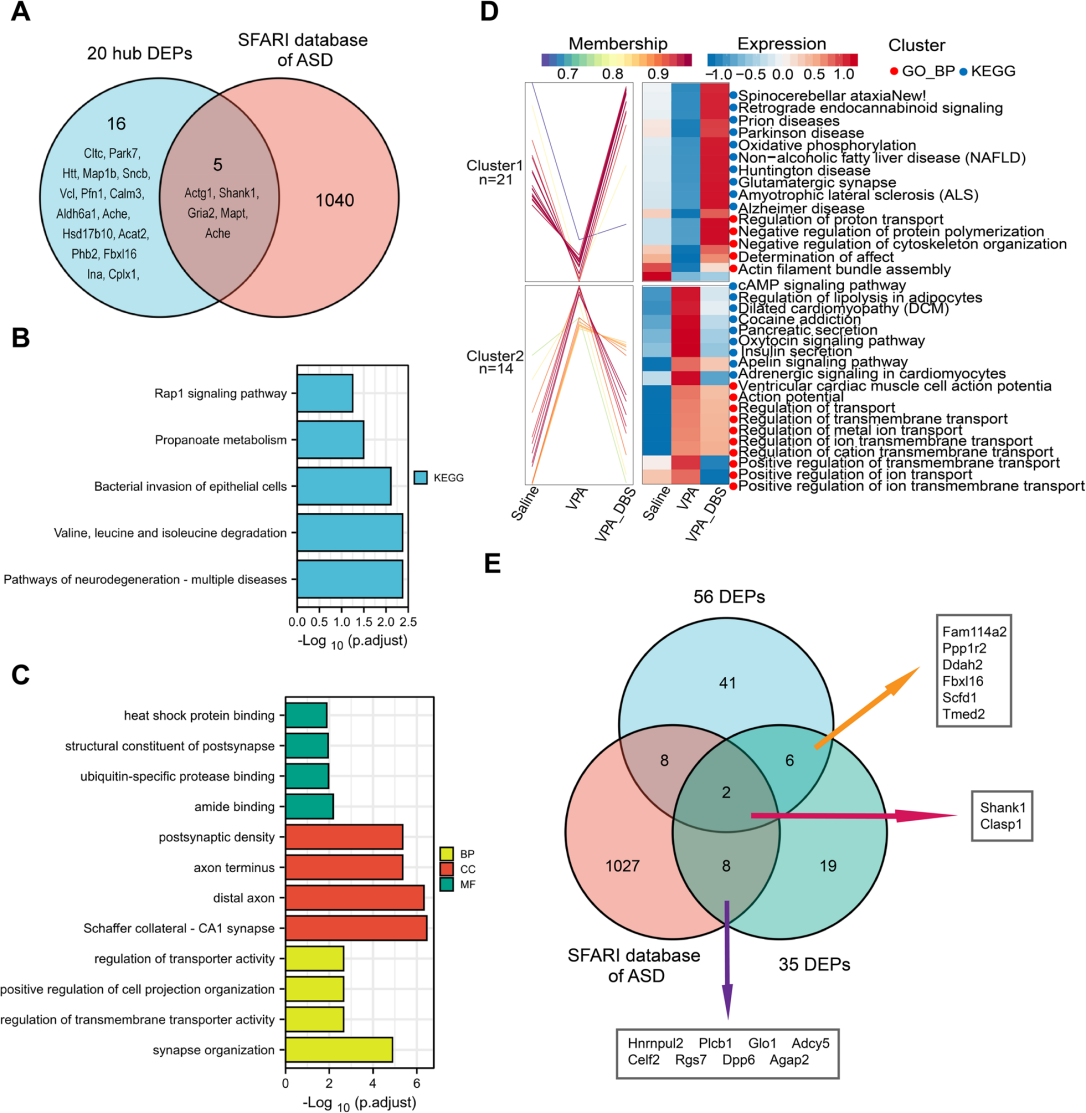
**A** Venn diagram of 20 hub DEPs and SFARI gene database. **↑**, upregulated; **↓**, downregulated. **B** KEGG pathways screened among 20 hub proteins. **C** GO analysis of 20 hub proteins. **D** Cluster map of Mfuzz expression patterns. The left side of the figure is a line graph of protein expression, and the right side is a heat map of protein expression. Each cluster corresponds to a line graph and a heatmap. The kegg pathway and GO enrichment information are shown to the right of the corresponding heatmap. **E** Venn diagram among DEPs of VPA versus saline batches (56 DEPs), DEPs after DBS intervention (35 DEPs) and the SFARI gene database. *KEGG* Kyoto Encyclopedia of Genes and Genomes, *GO* Gene Ontology

The below proteins of interest were subjected to further analysis: hub proteins as well as the gene that codes for DEPs belonging to genes associated with autism. Twenty hub proteins originating from 56 different DEPs were detected based on the STRING database and Cytohubba's analysis, including MAPT, ACTG1, CLTC, PARK7, HTT, GRIA2, MAP1b, SNCB, VCL, SHANK1, PFN1, CALM3, ALDH6A1, HSD17B10, ACHE, INA, CPLX1, ACAT2, PHB2, and FBXL16 (Fig. 6G). In addition, referring to the genes associated with autism shown in the SFARI (Simons Foundation Autism Research Initiative) gene database (https://gene.sfari.org/), ten genes were screened out encoding DEPs (ACTG1, GSTM1, SHANK1, GRIA2, PPP5C, MAPT, ClASP1, CLTC, ACHE, and SLC4A10) that were included in the 56 DEPs, which implies commonalities in the interplay between genetic and environmental factors. Table S1 has been annotated with the aforementioned two categories of DEPs. There were five DEPs whose coding genes were associated with autism that corresponded to the hub proteins (Fig. 7A). Notably, the KEGG analysis illustrated that 20 hub proteins were primarily associated with pathways of neurodegeneration-multiple disease and valine, leucine, and isoleucine degradation (Fig. 7B). They also mainly belonged to the distal axon, Schaffer collateral -CA1 synapse, axon terminus, and postsynaptic density participating in the biological process of synapse organization. (Fig. 7B).

## Differential expression protein analysis after the HF-DBS intervention in the VPA-exposed rats

To further understand how the differentially expressed proteins changed after the HF-DBS intervention, we first analyzed all significant proteins in the saline, VPA, and VPA-DBS batches and performed an expression pattern clustering analysis using the Mufzz method. We found 35 differentially expressed proteins, of which 21 were down-regulated in VPA batches compared with saline batches and up-regulated after the HF-DBS intervention (Cluster 1), and the remaining 14 differential proteins were up-modulated in the VPA batches and down-regulated after the HF-DBS intervention (Cluster 2) (Table 1). Next, The most substantially enriched entries in the KEGG pathway and GO enrichment analysis findings (biological process) are displayed on the right side of the expression profile clustering graph (Fig. 7D). Cluster 1 contains proteins strongly up-modulated in the saline and VPA-DBS batches that are implicated in, the negative modulation of protein polymerization, the modulation of proton transport, the negative modulation of cytoskeleton organization, and the determination of its effects, and the actin filament bundle assembly related in GO terms. In KEGG pathway terms, proteins strongly up-regulated in the saline and VPA-DBS batches that are involved in spinocerebellar ataxia, non-alcoholic fatty liver disease (NAFLD), retrograde endocannabinoid signaling, amyotrophic lateral sclerosis (ALS), Parkinson's disease (PD), Huntington's disease (HD), prion diseases, glutamatergic synapse, oxidative phosphorylation, and Alzheimer’s disease (AD). Cluster 2 contains proteins down-regulated in the saline and VPA-DBS batches that are involved in ventricular cardiac muscle cell action potential, action potential, positive modulation of ion transport, modulation of transmembrane transport, positive modulation of transmembrane transport, modulation of ion transmembrane transport, modulation of metal ion transport, modulation of cation transmembrane transport, modulation of transport, positive modulation of ion transmembrane transport-related GO terms. In KEGG pathway terms, proteins down-regulated in saline and VPA-DBS batches that are involved in the cAMP signaling pathway, modulation of lipolysis in adipocytes, dilated cardiomyopathy (DCM), cocaine addiction, pancreatic secretion, oxytocin signaling pathway, insulin secretion, circadian entrainment, apelin signaling pathway, adrenergic signaling in cardiomyocytes. Next, we further screened out ten genes encoding DEPs (HNRNPUL2, PLCB1, GLO1, ADCY5, CELF2, RGS7, DPP6, SHANK1, CLASP1, and AGAP2) from the SFARI gene database that also included 35 DEPs and two of the DEPs (SHANK1 and FBXL16) belonged to the hub proteins screened out from the 56 DEPs (Table 1). Two of the DEPs were co-identified in three types of DEPs (56 DEPs, 35 DEPs, and the SFARI database of ASD). Within the group of 35 DEPs, there were eight DEPs whose coding genes were linked to autism; six of the DEPs from 35 DEPs also belonged to 56 DEPs (Fig. 7E). The above results suggested that a potential way in which HF-DBS could improve the VPA-exposed rats’ phenotypes would be to normalize protein expression in the AI.

**Table 1:** The list of 35 DEPs in expression pattern clustering analysis after DBS intervention

Protein ID	Gene name	Protein name	Cluster	Membership
A9UMV9	Ndufa7	Complex I-B14.5a	Cluster1	0.9920
P62329	tmsb4x	Thymosin beta-4	Cluster1	0.9918
F1LT49	Lrrc47	Leucine-rich repeat-containing 47	Cluster1	0.9916
Q6MG60	Ddah2^*^	N(G),N(G)-dimethylarginine dimethylaminohydrolase 2	Cluster1	0.9891
D4ABT8	Hnrnpul2^b^	Heterogeneous nuclear ribonucleoprotein U-like 2	Cluster1	0.9971
B0K020	Cisd1	CDGSH iron-sulfur domain-containing protein 1	Cluster1	0.9975
Q63524	Tmed2^*^	Transmembrane emp24 domain-containing protein 2	Cluster1	0.9882
P10687	Plcb1^b^	1-phosphatidylinositol 4,5-bisphosphate phosphodiesterase beta-1	Cluster1	0.9976
G3V864	plppr4	Phospholipid phosphatase-related protein type 4	Cluster1	0.9873
D4A5L9	LOC690675	Similar to cytochrome c, somatic	Cluster1	0.9985
P13668	Stmn1	Stathmin	Cluster1	0.9790
D3ZC89	Fam114a2^*^	Family with sequence similarity 114, member A2	Cluster1	0.9785
Q6P7Q4	Glo1^b^	Lactoylglutathione lyase	Cluster1	0.9784
Q5RKG9	Eif4b	Eukaryotic translation initiation factor 4B	Cluster1	0.9953
A0A0H2UHA0	Ppp1r2^*^	Protein phosphatase inhibitor 2	Cluster1	0.9942
F1M4R7	Cspg5	Chondroitin sulfate proteoglycan 5	Cluster1	0.9465
P20788	Uqcrfs1	Cytochrome b-c1 complex subunit Rieske, mitochondrial	Cluster1	0.9762
D3ZU64	Vps37b	VPS37B subunit of ESCRT-I	Cluster1	0.9796
Q9WV48	Shank1^*ab^	SH3 and multiple ankyrin repeat domains protein 1	Cluster1	0.6440
F1M820	Sorbs1	Sorbin and SH3 domain-containing protein 1	Cluster1	0.9929
A0A0G2JSM7	Add1	Adducin 1 (Alpha), isoform CRA_b	Cluster1	0.9942
G3V9G1	Adcy5^b^	Adenylate cyclase type 5	Cluster2	0.9056
D3ZAP9	Gpd1l	Glycerol-3-phosphate dehydrogenase [NAD( +)]	Cluster2	0.8912
Z4YNP1	Celf2^b^	CUGBP Elav-like family member 2	Cluster2	0.9632
Q3S4A5	Arfgap1	ADP-ribosylation factor GTPase activating protein 1 brain isoform	Cluster2	0.9142
Q5MJ12	Fbxl16^*a^	F-box/LRR-repeat protein 16	Cluster2	0.9737
B0LPN4	Ryr2	Ryanodine receptor 2	Cluster2	0.9860
D3ZWG2	Rgs7^b^	Regulator of G-protein-signaling 7	Cluster2	0.8349
Q62991	Scfd1^*^	Sec1 family domain-containing protein 1	Cluster2	0.8875
F1LMR7	Dpp6^b^	Dipeptidyl aminopeptidase-like protein 6	Cluster2	0.7682
D3ZMJ7	Wnk2	Non-specific serine/threonine protein kinase	Cluster2	0.9916
Q63525	Nudc	Nuclear migration protein nudC	Cluster2	0.9241
A0A0G2JTD7	Clasp1^*b^	Cytoplasmic linker-associated protein 1	Cluster2	0.8811
D3ZT36	Adam23	ADAM metallopeptidase domain 23	Cluster2	0.8691
Q8CGU4	Agap2^b^	Arf-GAP with GTPase, ANK repeat and PH domain-containing protein 2	Cluster2	0.9909

^*^Proteins could be normalized after HF-DBS intervention that also belong ro the 56 DEPs obtained from VPA versus saline batches

^a^20 hub DEPs from 56 DEPs

^b^SFARI database of ASD

*DEPs* differentially expressed proteins

## Discussion

In the present investigation, new experimental data suggesting that DBS in autism might be a viable treatment option has been revealed. In short, we have systematically examined the influence of HF-DBS on autism-like behaviors and demonstrated that this approach improves sociability and repetitive/stereotypic-like behaviors. The optimal effects of autism-like behaviors were achieved after seven days of stimulation and these phenotypes were still improved after one week when the stimulation ceased. Unfortunately, we have not observed the exact effects on spatial learning and cognitive rigidity in the VPA-exposed test subjects. We found that HF-DBS may cause a slight increase in distance traveled, which may be related to the increase in exploratory behavior. In addition, the duration of self-grooming behavior was decreased without a change in self-grooming numbers.

In this research, we first implanted electrodes into rats that had been subjected to valproic acid treatment to cause autism, and then we stimulated the animals thereafter. In addition, we used a novel design informed by prior research with an implantable stimulation generator, which allowed the test subjects to move and eat unrestrictedly and provide long-term chronic stimulation (effective voltage output for three months) [36]. We have observed that the expression levels of the Npas4, Arc, and c-Fos genes in the AI of the VPA-exposed animals were considerably elevated as opposed to that of rats in the Saline group, which may be related to abnormal responses to neuronal activity in neural circuits [31, 37]. Interestingly, long-term HF-DBS increased the expression of the IEGs in the AI of the saline-DBS group, and conversely reduced the expression of IEGs in the VPA-DBS group, suggesting that our DBS protocol dynamically modulated the AI neuronal activity, and this is consistent with previous studies [36, 38, 39].

The insula, as a key part of the “salient network”, has afferent or efferent connections with multiple brain regions, such as the motor cortex, sensory cortex, prefrontal cortex, nucleus accumbens, striatum, and midbrain, among others, which is considered a central hub of interoception and mediates the processing of external and bodily sensory information [15], bodily and self-awareness [40], emotion regulation [41], complex social-affective functions, and switches among large-scale brain networks [22, 31]. In preclinical studies, anterior insula neurons encode social exploration behaviors and are associated with reward processing, impulsive decision making, and compulsive behavior [21, 42, 43]. The optogenetic silencing of the insular cortex prevents social affective preferences and oxytocin alters the excitability of the insular cortex to regulate social and asocial responses [22]. The above findings suggest that the insula mediates the processing associated with autism-like behaviors. Due to the extensive fiber connections to other brain regions as well as complex social, emotional, and sensory functions of the anterior insula, we explored the possibility of DBS in this region to intervene in autism-like behaviors. In addition to significant social impairment, increased repetitive stereotypes and lowered pain sensitivity was also observed in our study, which highly overlaps with ASD features [23, 44–46]. Meanwhile, we also observed weakened spatial learning ability and marked cognitive rigidity in animals. Previous studies have revealed that HF-DBS applied to the infralimbic prefrontal cortex diminished hyperlocomotion and improved social interaction in olfactory bulbectomized (OBX) rats and the VPA-induced rat model [12, 47]. HF-DBS at the central thalamus can restore social interaction via the enhancement of the corticolimbic and corticostriatal circuits [13]. Furthermore, in both ASD mouse models Shank3B^−/−^ and Viaat-Mecp2^−/y^, HF-DBS inhibited self-grooming at the subthalamic nucleus, and HF-DBS in both the internal capsule and striatum reduced excessive grooming in Sapap3 mutant mice [14, 48]. In this study, hyperlocomotion was not observed in adult VPA-exposed rats, which is consistent with earlier research findings [23]. We discovered that HF-DBS improved sociability and social novelty preferences in VPA-treated animals, while the duration of self-grooming was reduced and increased exploration time in the inner zone of the open-field experiment was reduced. Therefore, stimulation in the anterior insula may alter the connectivity of the prefrontal cortex and thalamus, specifically causing a block in axonal conduction and reducing the hyperexcitability of downstream neurons [49, 50]. At the same time, stimulation may lead to the neurotransmitter depletion of axonal terminal synapses and weakened synaptic conduction [51]. These complex combined effects are involved in the modulation of social behaviors and cognitive functions in VPA-exposed rats. Previous studies have shown that VPA-exposed animals and related autism-like models present learning and cognitive dysfunction [35, 46, 52], which may relate to neuronal degeneration and apoptosis in brain areas, including the hippocampus, striatum, and prefrontal cortex [53, 54]. Our study also found impaired spatial learning abilities and cognitive rigidity in the VPA-exposed group, but HFS-DBS failed to ameliorate this impairment, possibly due to less bidirectional output from the hippocampus and insula [16]. The insula may be more involved in emotional and interoceptive processing than learning and memory [54].

Based on the revelations of HF-DBS in AI on autism-like behaviors, we further explored its possible mechanisms and key proteins through proteomic analysis. As per the findings of our proteomic research, the expression levels of 56 proteins in AI are substantially different in the VPA-exposed group in contrast with the saline-exposed group. From the biological process analysis, the down-regulated DEPs were mainly related to positive modulation of axon extension, positive modulation of microtubule polymerization, and microtubule cytoskeleton organization, including MAPT, MAP1B, TRPV2, and CALM3. From the cellular component analysis, four DEPs were found in presynapse, including SNCB, ATP2B3, PARK7, and CPLX1; five DEPs were found in neuron projection, including PARK7, CALM3, PPP5C, HNRNPA3, SHANK1. Synaptic dysfunction is one of the pathogeneses of ASD and numerous psychiatric disorders, and the maintenance of synaptic homeostasis is essential to ensuring that the central nervous system (CNS) continues to function normally, whereby dendrites and axons are closely related to synapse formation [55]. Many ASD risk genes encode microtubule-related proteins that influence the strength and number of synapses and eventually influence neuronal projections in the brain [55, 56]. KEGG analysis showed that, in up-regulated DEPs, synaptic vesicle cycle and lysosome pathways were significantly enriched. These pathways both involved 2 DEPs, including CLTC and ATP6V0A1. CLTC is known to encode clathrin heavy chain 1 (CHC1), which is expressed at a high level in the developing brain and might perform an integral function in neuronal transmission by promoting the recycling and/or release of neuronal presynaptic terminal vesicles [57]. Previous studies have shown that CLTC variants are linked to different phenotypes ranging from mild to severe intellectual disability, epilepsy, corpus callosum hypoplasia, and microcephaly [58]. ATP6V0A1 encodes V-type proton ATPase subunit a, which is a significant factor for the vacuolar proton pump (V-ATPase), an enzyme with many subunits that is responsible for facilitating the movement of protons between membranes. Lysosome homeostasis plays an important role in neurodevelopment and behavior [59, 60]. ATP6V0A1 variants may lead to lysosomal dysfunction, further contributing to cell death, the autophagosome, and lysosomal accumulation, while attenuating synaptic connectivity and mTORC1 signaling in addition to reducing neurotransmitter content in synaptic vesicles [61]. As for the down-modulated DEPs, two pathways were significantly enriched in valine, leucine, and isoleucine degradation, and neurodegeneration-multiple diseases, respectively. The former includes ALDH3A2, ALDH6A1, and ACAT2, the latter includes MAPT, GRIA2, HTT, PARK7, and CALM3. ALDH3A2 gene encodes fatty aldehyde dehydrogenase (FALDH; EC 1.2.1.48), which is an enzyme responsible for catalyzing the oxidation of the fatty aldehyde to the fatty acid. ALDH6A1 gene encodes methylmalonate semialdehyde dehydrogenase (MMSDH; EC:1.2.1.27). The above two belong to the aldehyde dehydrogenase (ALDH) gene family. FALDH assumes a fundamental function in lipid metabolism and is prominent in skin function and neurodevelopment. Defects in the ALDH3A2 gene can lead to Sjögren-Larsson syndrome (SLS), patients who are afflicted with this condition develop spastic diplegia and mental impairment, as well as ichthyosis [61]. Previous studies using metabolic profiles derived from proton nuclear magnetic resonance spectroscopy revealed that levels of valine, leucine, and isoleucine in the hippocampus were increased in a VPA-triggered rat model, which may be interpreted by the reduction of methyl malonate [62]. There have been reports of similar disruptions in the levels of these amino acids in the urine of children with autism as well as in the blood of children with propionic acidosis and autistic characteristics [63, 64]. In our study, MMSDH (encoded by the ALDH6A1 gene) was significantly downregulated in the insula of VPA batches, further explaining the above results.

Depending on the outcomes of the assessment of the hub protein and the comparison of this data with the ASD gene database, the following DEPs might perform an instrumental function in the pathogenic mechanisms of VPA-exposed rats: including MAPT, ACTG1, CLTC, PARK7, HTT, GRIA2, SNCB, MAP1B, SHANK1, and ACHE, which have largely overlapped with the DEPs in the neurodegeneration-multiple diseases pathway (MAPT, GRIA2, HTT, PARK7, and CALM3). Members of the neuronal microtubule-associated proteins (MAPs) family have been shown to maintain microtubules, modulate microtubule networks in dendrites and axons, and interact with various proteins while being connected to ASD [55]. The microtubule-associated protein 1B (MAP1B) and microtubule-associated protein tau (MAPT) are the predominant members of MAPs, with MAPT being most abundant in neuronal axons and MAP1B is uniformly dispersed in axons, cell bodies, and dendrites of growing nerve cells [65]. MAPT and MAP1B perform an integral synergistic role in neuronal migration and axonal elongation, hypoplasia of commissural axonal tracts, and it was discovered that the neuronal layering in the brains of *tau*^+/+^ *map1b*^–/–^ mice was disordered with the *tau*^–/–^*map1b*^–/–^ double mutants exhibiting extremely severe phenotypes [66]. Our proteomic results also showed downregulation of MAPT and MAP1B in the AI of VPA batches, which may lead to impaired axonal and synaptic function. Although previous studies have suggested that tau reduction prevents key features of autism in Cntnap2^–/–^ and Scn1a^RX/+^ mice, this may not contradict our findings [67]. The interaction of tau with many other proteins results in an unfavorable gain of function, which is associated with the formation of aberrant tau structures and assemblies. Complex environmental and genetic factors may lead to compensatory changes in the structure or number of tau. Actin is an essential cellular protein involved in many important functions in various systems of the body, including cell motility, cytokinesis, structural support, and regulation of gene expression [68]. In the nervous system, these functions are mainly undertaken by beta-actin (encoded by ACTB) and gamma-actin (encoded by ACTG1) [69]. The cytoplasmic actin gene sequences Actb and Actg1 are highly conserved and very similar, respectively [70]. Mutations of the two often lead to deafness and developmental disorders, such as intellectual disabilities and growth retardation [71]. Here, actin encoded by Actg1 is downregulated in the AI, which may contribute to cognitive impairment in VPA-exposed rats. Huntingtin is thought to be a key protein of the devastating neurodegenerative disorder Huntington’s disease (HD), which is critical for microtubule-mediated transport, vesicle function, autophagy, and transcription [72]. Huntingtin is encoded by the HTT gene, and its structural abnormality often results in loss of functionality and an enhancement in HD risk [73]. A study found that *Htt*-KO mice exhibited autism-like behavioral characteristics like *Fmr1*-KO mice and that the mitochondrial fusion phenotype could be rescued by enhancing the *Htt* expression [74]. The present study found a decreased HTT in the AI of VPA rats, which is congruent to the findings of an earlier study [75]. The results suggested that the downregulation of HTT may lead to abnormal behavior in VPA-exposed rats. Acetylcholinesterase (AchE), as a special carboxylate hydrolase, can hydrolyze the neurotransmitter acetylcholine at the brain cholinergic synapses and neuromuscular junctions, thereby terminating signal transduction, and it assumes an important role in neuronal apoptosis [76]. A positron emission tomography study showed decreased acetylcholinesterase activity in the fusiform gyrus in adults with autism spectrum disorder [77]. Our study found that AchE is also downregulated, which may affect normal neural signal transduction.

Indeed, among the up-modulated DEPs, three (DLGAP4, INA, and PFN1) and one (PHB2) are associated with glutamatergic and GABAergic synapses, correspondingly. As for the down-modulated DEPs, four (ATP2B3, CPLX1, SHANK1, and HTT) and one (ATP2B3) are associated with glutamatergic and GABAergic synapses, correspondingly. In addition, PARK7, SHANK1, and GRIA2 are involved in the negative modulation of the activity of the N-methyl-d-aspartate receptor (NMDAR) and ionotropic glutamate receptor function. Glutamate is the predominant central excitatory neurotransmitter in the brain, and it also has the potential to serve as a precursor to a variety of other substances, including the primary inhibitory neurotransmitter gamma-aminobutyric acid (GABA) [78, 79]. GABAergic inhibition is central to shaping the complex input–output and plastic characteristics of neural networks, establishing a homeostatic process that sustains the network's excitability and plasticity at appropriate levels to allow the gating, processing, and storage of information [80]. It performs a pivotal function in a variety of developmental mechanisms including cell proliferation, migration, differentiation, synapse maturation, neural connection, and others [81]. Glutamatergic and GABAergic synapses are involved in excitatory/inhibitory (E/I) balance, and diverse psychiatric and neurological disorders, especially autism, have been associated with alterations in GABAergic signaling in particular neuronal circuits and also disrupt E/I equilibrium [80–82]. E/I imbalances have been identified in numerous brain regions in VPA-exposed rats, including the hippocampus, temporal cortex, and prefrontal cortex [83, 84]. In summary, the cognitive impairment and autism-like behavior of the VPA rats might be attributable to the dysregulation of synaptic and associated receptor proteins in the AI of these rats.

Our proteomic analysis reveals that 35 DEPs in AI were normalized by DBS interventions. In cluster1, KEGG analysis showed that the DEPs after DBS intervention were mainly concentrated in multiple neuropsychiatric disease pathways, including PD, HD, ALS, and AD, which involved the DEPs encoded by four genes (NDUFA7, LOC690675, PLCB1, and UQCRFS1). Besides, NDUFA7 and UQCRFS1 were also enriched in the oxidative phosphorylation pathway, while PLCB1 and SHANK1 were enriched in the glutamatergic synapse. Phospholipase C-beta 1 (encoded by PLCB1) induces intracellular signaling downstream of G protein-coupled receptors, and activation of PLCB1 by metabotropic glutamate receptor 5 (mGluR5) is integral for the coordinated development of presynaptic and postsynaptic elements [85, 86]. In patients with ASD, the gene expression level of mGluR5, as well as its downstream signaling protein PLCB1, was shown to be considerably decreased across all age groups, according to results from a previous microarray investigation, which is in line with our findings [87]. Meanwhile, in the present study, DBS could restore the protein expression of PLCB1, making it involved in coordinating the normal function of glutamate synapses. The above-mentioned DEPs are related to mitochondrial respiration, oxidative phosphorylation, and synaptic function, indicating that the signaling pathways and processes underlying these disorders are similar, which can help us better understand their etiology and DBS treatment mechanisms. In cluster 2, two DEPs were concentrated in multiple signaling pathways, such as circadian entrainment, apelin signaling pathway, oxytocin signaling pathway, and cAMP signaling pathway, which are encoded by RYR2 and ADCY5. A class of enzymes known as adenylyl cyclase type 5 (AC5) is responsible for the production of cyclic AMP (cAMP) from ATP when neurotransmitter receptors are activated, thereby regulating signaling downstream of coupled receptors [88]. It is a crucial effector for a wide variety of neurotransmitter receptors, particularly glutamate and dopamine receptors [88, 89]. However, it should be mentioned that loss of AC5 in the dorsal striatum produces autism-like behaviors [90]. On the other hand, the expression level of AC5 was shown to be elevated in the insula of VPA rats in this investigation, as was described previously. DBS intervention can restore this abnormal change.

Although we obtained 35 DEPs after DBS intervention, we could not screen out the concentrated hub proteins, which shows that the mechanism of action of DBS is complex and extensive. SHANK1 and CLASP1 were co-identified in three types of DEPs, which may perform a key function in the pathogenic mechanism of autism and the therapeutic effect of DBS. Mutations in SH3 and multiple ankyrin repeat domains protein (SHANK) family genes are linked to syndromic and idiopathic ASD, as well as to other neuropsychiatric disorders [91–93]. SH3 and multiple ankyrin repeat domain proteins 1, also abbreviated as SHANK1, can serve as an adapter protein that may be found in the postsynaptic density of excitatory synapses, which connects NMDA-type and metabotropic glutamate receptors [90]. A study showed that SHANK1 is responsible for modulating excitation synaptic transmission in inhibitory interneurons in addition to its role in controlling excitatory synaptic transmission neurons, thereby altering the E/I equilibrium in local brain regions [94, 95]. Earlier research reports have illustrated that SHANK1 is implicated in acoustic communication across species, and SHANK1 gene deletion contributes to deficits in social communication/interactions. SHANK1 and SHANK3 double knockout mice result in repetitive behaviors and social deficits, while also showing deficits in learning and motor behaviors. In agreement with earlier research, this study found that SHANK1 was significantly downregulated in the insula of VPA rats, and DBS intervention could reverse it, which may be an important part of improving behavioral abnormalities.

This research does have certain shortcomings that need to be addressed. First, this study explored the impacts of different cycles of HF-DBS on autism-like animal models, however, the potential of low frequency-DBS (LF-DBS) or an integration of both HF- and LF-DBS to influence the development of autism-like behaviors warrants additional investigation. Furthermore, the protein samples were taken from the rat insula tissue after 14 days of DBS stimulation, but the changes of protein in the insula after early stimulation and stimulation cessation need to be explored. Finally, only male animals were selected for the experiment in this study. Epidemiological studies have illustrated that ASD is more common in men than in women, and ratios of 2:1 up to 5:1 have been recorded [1]. According to the findings of a prior investigation, diagnosed females, in general, had a lower level of restricted/repetitive/behavior/interests (RRBI) compared to men [96]. Both estrogen and the related receptors are broadly distributed throughout the brain, and there is growing evidence that disorders of estrogen and estrogen signaling pathways are involved in neuropsychiatric illnesses, including ASD [97]. Estrogen receptors (ER) and estrogen signaling pathways have a neuroprotective role in cognitive function by activating numerous nervous systems, including glutamatergic, serotonergic, and dopaminergic systems [98, 99]. In a recent study, it was shown that both the protein and mRNA levels of estrogen receptor beta (ER-beta) were decreased in the middle frontal gyrus of the postmortem brains of ASD patients. A disruption in ER coactivators was also shown to be present in individuals with ASD who were involved in the same research. Patients with ASD were also found to have lower levels of CREB-binding protein (CBP) and steroid receptor coactivator-1 [99]. Further, analysis of the levels of serum G-protein-coupled estrogen receptor (GPER) demonstrated that GPER levels were substantially reduced in ASD patients [100]. The above results illustrate that gender and hormonal status may influence the developmental process of ASD, and gender factors deserve to be distinguished in related research. Thus, it is necessary to conduct additional research on the impact that HF-DBS has on autistic female rats.

## Conclusion

In summary, our findings illustrated that continuous HF-DBS in bilateral AI can improve sociability and repetitive/stereotypic-like behaviors in VPA-exposed rats by reversing the expression of autism-related proteins. But it does not affect the spatial learning ability and cognitive rigidity of animals. In light of past research as well as the findings of this study, the insula may be a potential target for DBS in the treatment of autism, which provide a theoretical basis for its clinical application.

## Supplementary Information

**Additional file 1: Figure S1.** The social ability of animals was not affected by the way of electrode implantation. **(A) **Time spent by test rats in Chamber S1. **(B)** Time spent by test rats in Chamber S2. **(C)** Sniffing time between test rats and S1. **(D)** Sniffing time between test rats and S2. **(E)** Sociability index. **(F)** Social novelty preference index. **(G)** Representative heat maps of three-chamber social interaction test on day 18. Data are shown as mean with SD. Two-way repeated ANOVA with post-hoc Bonferroni test, Sample sizes (n): saline, n = 13; saline-sham, n = 13; VPA, n = 12; VPA-sham, n = 12.

**Additional file 2: Figure S2.** The repetitive/stereotypic-like activities were not affected by the way of electrode implantation. **(A)** Total distance travelled by test rats. **(B)** Number of self-grooming behavior. **(C)** Duration of self-grooming behavior. **(D) **Number of marbles buried. Data are shown as mean with SD. **(E) **The representative marbles buried maps of marbel burying test on day 18. Two-way repeated ANOVA with post-hoc Bonferroni test. Sample sizes (n): saline, n = 13; saline-sham, n = 13; VPA, n = 12; VPA-sham, n = 12.

**Additional file 3: Table S1. **The list of 56 DEPs detected in VPA versus saline batches.

## Data Availability

The datasets used and/or analysed during the current study are available from the corresponding author on reasonable request.

## Abbreviations

ASD: Autism spectrum disorder
VPA: Valproic acid
DBS: Deep brain stimulation
AI: Anterior insula
IPG: Implantable pulse generators
RT-qPCR: Real-time quantitative polymerase chain reaction
DEP: Differential expression protein
KEGG: Kyoto encyclopedia of genes and genomes
GO: Gene ontology
BP: Biological process
CC: Cellular component
MF: Molecular function
PPI: Protein-protein interaction
