# Supplementary figures and images for: Continuous high-frequency deep brain stimulation of the anterior insula modulates autism-like behavior in a valproic acid-induced rat model
Source: J Transl Med. 2022 Dec 6;20:570. doi: 10.1186/s12967-022-03787-9 (PMC9724311; doi:10.1186/s12967-022-03787-9)

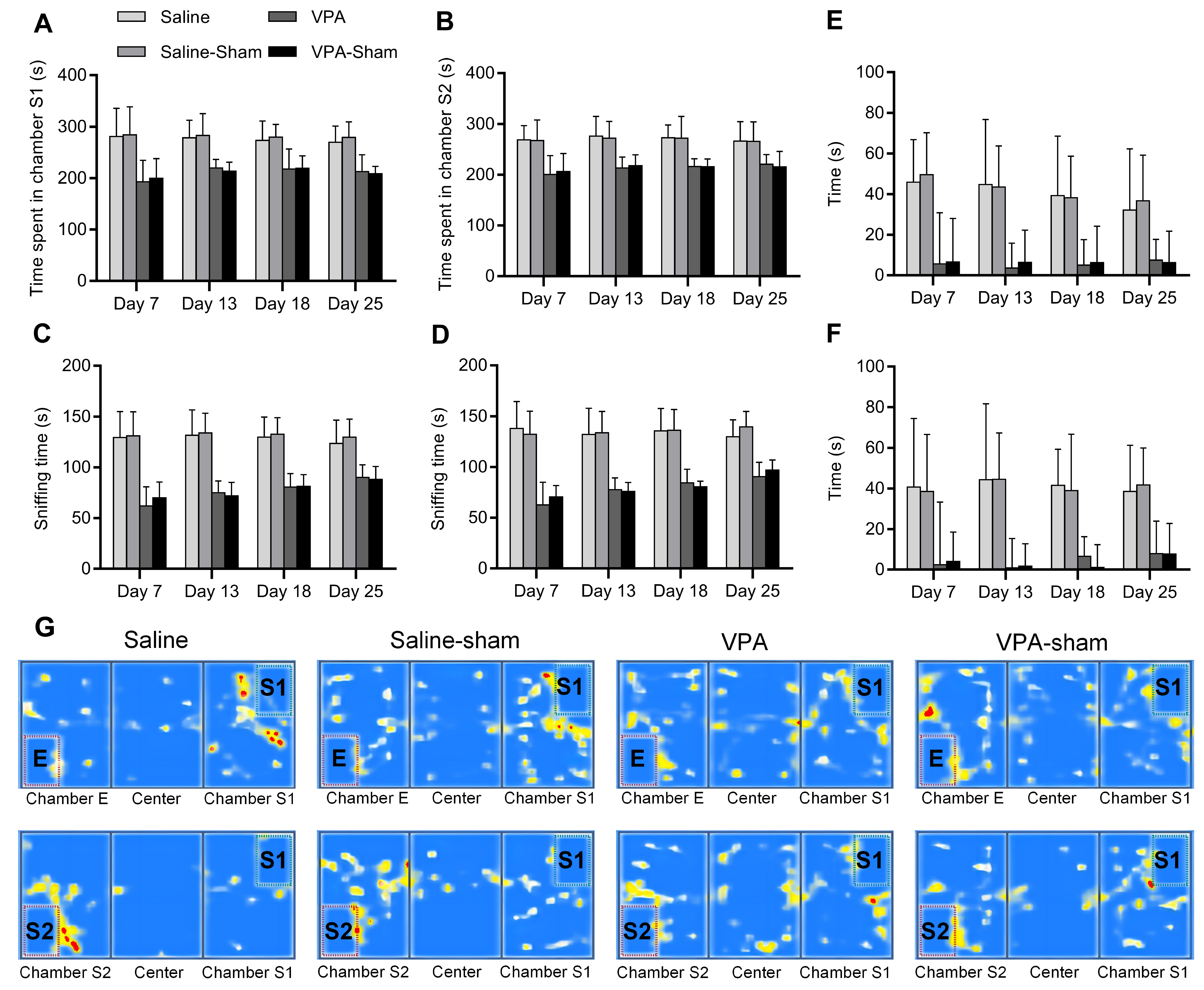

Supplement: Supplementary file 1 — Additional file 1: Figure S1. The social ability of animals was not affected by the way of electrode implantation. (A) Time spent by test rats in Chamber S1. (B) Time spent by test rats in Chamber S2. (C) Sniffing time between test rats and S1. (D) Sniffing time between test rats and S2. (E) Sociability index. (F) Social novelty preference index. (G) Representative heat maps of three-chamber social interaction test on day 18. Data are shown as mean with SD. Two-way repeated ANOVA with post-hoc Bonferroni test, Sample sizes (n): saline, n = 13; saline-sham, n = 13; VPA, n = 12; VPA-sham, n = 12. [file 12967_2022_3787_MOESM1_ESM.png]

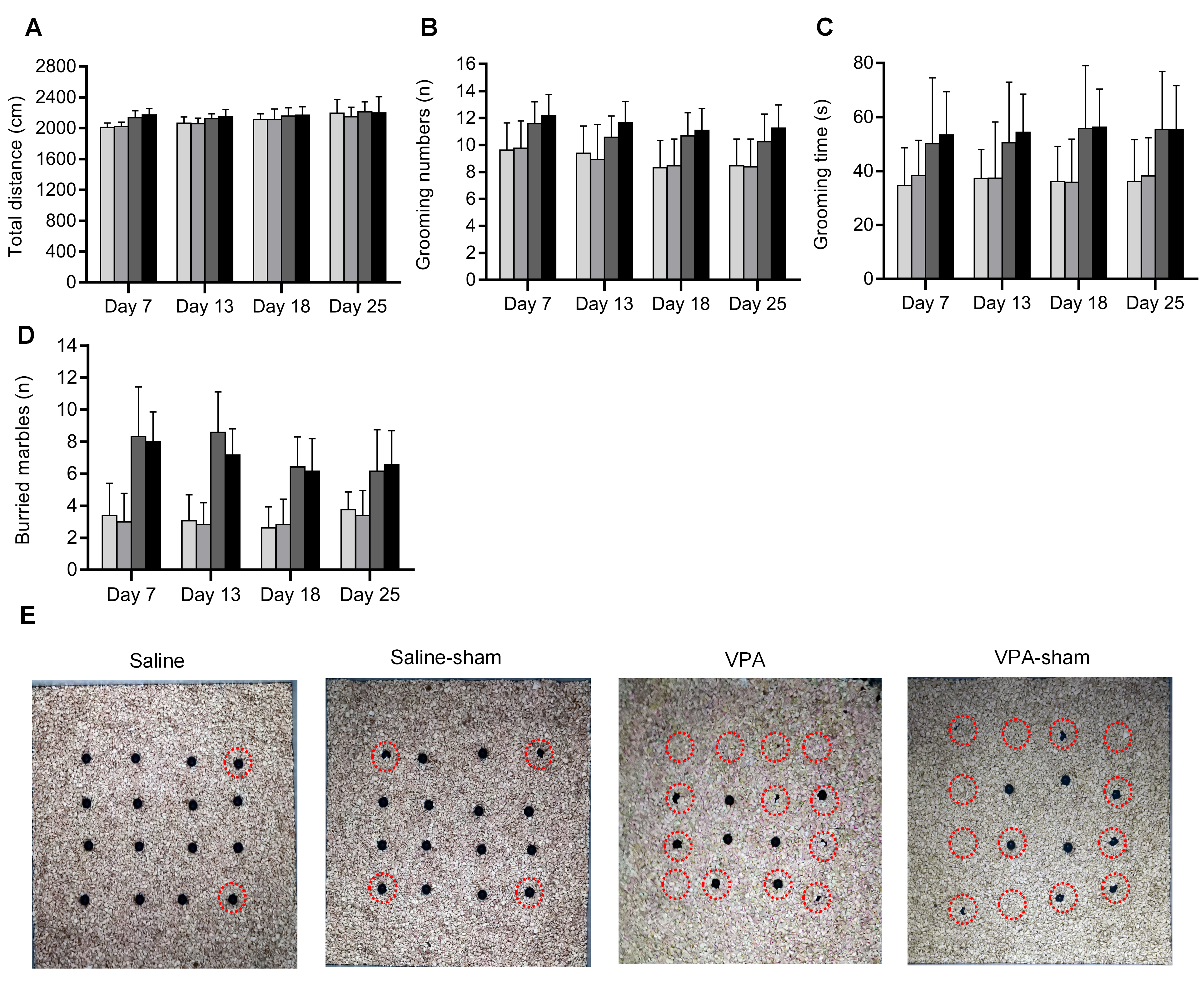

Supplement: Supplementary file 2 — Additional file 2: Figure S2. The repetitive/stereotypic-like activities were not affected by the way of electrode implantation. (A) Total distance travelled by test rats. (B) Number of self-grooming behavior. (C) Duration of self-grooming behavior. (D) Number of marbles buried. Data are shown as mean with SD. (E) The representative marbles buried maps of marbel burying test on day 18. Two-way repeated ANOVA with post-hoc Bonferroni test. Sample sizes (n): saline, n = 13; saline-sham, n = 13; VPA, n = 12; VPA-sham, n = 12. [file 12967_2022_3787_MOESM2_ESM.png]
