# Supplementary material for: Continuous high-frequency deep brain stimulation of the anterior insula modulates autism-like behavior in a valproic acid-induced rat model
Source: J Transl Med. 2022 Dec 6;20:570. doi: 10.1186/s12967-022-03787-9 (PMC9724311; doi:10.1186/s12967-022-03787-9)
Supplement: Supplementary file 3 — Additional file 3: Table S1. The list of 56 DEPs detected in VPA versus saline batches. [file 12967_2022_3787_MOESM3_ESM.docx]

Additional file

| **Table S1.** The list of 56 DEPs detected in VPA versus saline batches. | | | | | |
| --- | --- | --- | --- | --- | --- |
| Protein ID | Gene name | Protein name | Down/Up | Fold change | *P*-value |
| P0DP31 | Calm3^a^ | Calmodulin-3 | Down | 0.4893 | 0.0196 |
| P63259 | Actg1^ab^ | Actin, cytoplasmic 2 | Down | 0.6662 | 0.0060 |
| P04905 | Gstm1^b^ | Glutathione S-transferase Mu 1 | Down | 0.1245 | 0.0004 |
| M0R4A5 | Ache^ab^ | Carboxylic ester hydrolase | Down | 0 | 0.0002 |
| G3V9P7 | Htt^a^ | Huntingtin | Down | 0 | 0.0003 |
| R9PXU6 | Vcl^a^ | Metavinculin | Down | 0 | < 0.0001 |
| Q5U2P5 | C2cd2l | C2CD2-like | Down | 0 | < 0.0001 |
| A0A0H2UHZ2 | Nap1l4 | Nucleosome assembly protein 1-like 4 | Down | 0 | < 0.0001 |
| D3ZC89 | Fam114a2 | Uncharacterized protein | Down | 0 | 0.0005 |
| A0A0G2JSQ1 | Sncb^a^ | Beta-synuclein | Down | 0 | < 0.0001 |
| G3V7J0 | Aldh6a1^a^ | Aldehyde dehydrogenase family 6, subfamily A1, isoform CRA_b | Down | 0 | < 0.0001 |
| A0A0H2UHA0 | Ppp1r2 | Protein phosphatase inhibitor 2 | Down | 0 | 0.0002 |
| O88767 | Park7^a^ | Parkinson disease protein 7 homolog | Down | 0 | < 0.0001 |
| Q9WV48 | Shank1^ab^ | SH3 and multiple ankyrin repeat domains protein 1 | Down | 0 | 0.0003 |
| A0A0H2UHQ9 | Synpo | Synaptopodin | Down | 0 | < 0.0001 |
| F1LNE4 | Gria2^ab^ | Glutamate receptor | Down | 0 | < 0.0001 |
| A0A0G2JSH6 | Trpv2 | Transient receptor potential cation channel subfamily V member 2 | Down | 0 | < 0.0001 |
| Q5XI22 | Acat2^a^ | Acetyl-CoA acetyltransferase, cytosolic | Down | 0 | < 0.0001 |
| A0A0G2JY43 | Aldh3a2 | Aldehyde dehydrogenase family 3 member A2 | Down | 0 | 0.0021 |
| G3V9J7 | Rabep1 | Rab GTPase-binding effector protein 1 | Down | 0 | 0.000 |
| F1M471 | Epm2aip1 | EPM2A-interacting protein 1 | Down | 0 | 0.0001 |
| P53042 | Ppp5c^b^ | Serine/threonine-protein phosphatase 5 | Down | 0 | < 0.0001 |
| A0A0G2K9Q6 | Atp2b3 | Calcium-transporting ATPase | Down | 0.4902 | 0.0415 |
| P15205 | Map1b^a^ | Microtubule-associated protein 1B | Down | 0.5880 | 0.0280 |
| D4A1Q2 | Mapt^ab^ | Microtubule-associated protein | Down | 0.3773 | 0.0098 |
| Q6MG60 | Ddah2 | N(G),N(G)-dimethylarginine dimethylaminohydrolase 2 | Down | 0.3439 | 0.0130 |
| P63041 | Cplx1^a^ | Complexin-1 | Down | 0.5559 | 0.0481 |
| A0A0G2JVV5 | Usp14 | Ubiquitin carboxyl-terminal hydrolase | Down | 0.4657 | 0.0499 |
| Q6URK4 | Hnrnpa3 | Heterogeneous nuclear ribonucleoprotein A3 | Down | 0.5192 | 0.0325 |
| Q63041 | A1m | Alpha-1-macroglobulin | Up | 1.6184 | 0.0114 |
| Q63965 | Sfxn1 | Sideroflexin-1 | Up | 1.5996 | 0.0063 |
| D3ZWA8 | Appl1 | Adaptor protein, phosphotyrosine-interacting with PH domain and leucine zipper 1 | Up | Inf | 0.0177 |
| Q9QX69 | Lancl1 | Glutathione S-transferase LANCL1 | Up | Inf | 0.0049 |
| A0A1B0GWY5 | Arhgef2 | Guanine nucleotide exchange factor H1 | Up | Inf | 0.0002 |
| D4AAR7 | Ccdc136 | Coiled-coil domain-containing 136 | Up | Inf | 0.0004 |
| Q5MJ12 | Fbxl16^a^ | F-box/LRR-repeat protein 16 | Up | Inf | < 0.0001 |
| P62963 | Pfn1^a^ | Profilin-1 | Up | Inf | < 0.0001 |
| F1M392 | Limch1 | LIM and calponin homology domains 1 | Up | Inf | 0.0004 |
| G3V8L3 | Lmna | Lamin A, isoform CRA_b | Up | Inf | < 0.0001 |
| Q5U2U7 | Rnmt | mRNA cap guanine-N7 methyltransferase | Up | Inf | 0.0005 |
| Q62991 | Scfd1 | Sec1 family domain-containing protein 1 | Up | Inf | 0.0004 |
| G3V927 | Dlgap4 | Discs, large homolog-associated protein 4 (Drosophila) | Up | Inf | 0.0001 |
| A0A0G2JTD7 | Clasp1^b^ | Cytoplasmic linker-associated protein 1 | Up | Inf | 0.0004 |
| G3V7W7 | Anpep | Aminopeptidase | Up | Inf | 0.0013 |
| A0A0G2K6P5 | Mfsd4a | Major facilitator superfamily domain-containing 4A | Up | Inf | 0.0002 |
| Q6AXV4 | Samm50 | Sorting and assembly machinery component 50 homolog | Up | Inf | < 0.0001 |
| B0BMW2 | Hsd17b10^a^ | 3-hydroxyacyl-CoA dehydrogenase type-2 | Up | Inf | < 0.0001 |
| Q4G061 | Eif3b | Eukaryotic translation initiation factor 3 subunit B | Up | Inf | < 0.0001 |
| A0A0A0MXX1 | Akap7 | A-kinase anchor protein 7 isoforms delta and gamma | Up | Inf | < 0.0001 |
| Q63524 | Tmed2 | Transmembrane emp24 domain-containing protein 2 | Up | Inf | 0.0003 |
| P23565 | Ina^a^ | Alpha-internexin | Up | 2.4469 | 0.0211 |
| F1M779 | Cltc^ab^ | Clathrin heavy chain | Up | 1.6156 | 0.0396 |
| G3V741 | Slc25a3 | Phosphate carrier protein, mitochondrial | Up | 1.5282 | 0.0223 |
| Q2I6B2 | Atp6v0a1 | V-type proton ATPase subunit a | Up | 1.5651 | 0.0264 |
| A0A0G2JYF0 | Slc4a10^b^ | Anion exchange protein | Up | 1.6544 | 0.0406 |
| A0A0G2KB63 | Phb2^a^ | Prohibitin | Up | 1.6736 | 0.0216 |
| Up, upregulated; Down, downregulated. ^a^20 hub DEPs, ^b^SFARI database of ASD. DEPs: differentially expressed proteins. | | | | | |
